# A brief review on the palladium-catalyzed C–H activation reactions of 2-phenylpyridines

**DOI:** 10.1039/d5ra01203a

**Published:** 2025-04-08

**Authors:** Jesly Joy, T. D. Demina, Amruta Kush Durgi, Ajesh Vijayan

**Affiliations:** a Department of Chemistry, Christ University Bangalore India 560029 ajesh.vijayan@christuniversity.in

## Abstract

Transition metal-mediated C–H activation is a significant synthetic methodology, with palladium-catalysed C–H activation emerging as a powerful tool in organic synthesis. This review summarises the advances made by palladium-catalyzed C–H functionalisation reactions on the *ortho* position of 2-phenyl pyridine over the last two decades. The *ortho* position of 2-phenyl pyridine has been identified as a prime target for C–H activation due to its unique electronic and steric properties. This mild and selective transformation has enormous applications in the chemical field, such as drug discovery, natural products, agrochemical, and pharmaceutical industries. These highly regioselective, chemo-selective, eco-friendly reactions exhibit a wide substrate scope. This review accounts for the development of various palladium-catalyzed C–H functionalisation reactions of 2-phenyl pyridine.

## Abstract 2

This review highlights two decades of progress in palladium-catalyzed *ortho*-C–H functionalisation of 2-phenyl pyridine, showcasing its regioselectivity, broad scope, and relevance in pharmaceuticals, agrochemicals, and material science.

## Graphical abstract

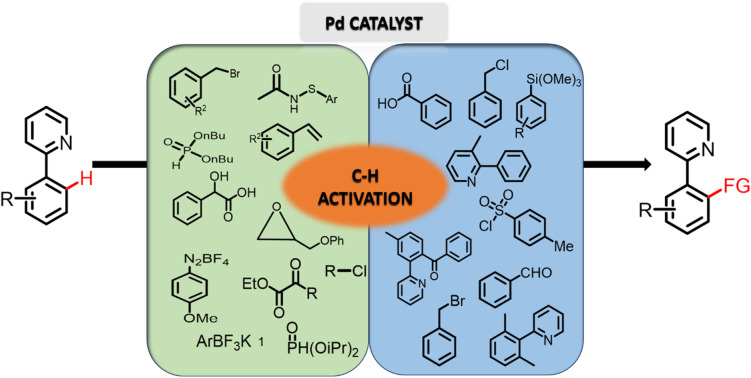

## 1. Introduction

The concept of environmentally sustainable and cost-effective synthesis has recently garnered significant attention.^1^ Over the last 15 years, the field of C–H activation has gained significant attention and is now recognized as a potentially useful technique for functionalizing organic compounds with applications in biology, materials science, and also in the pharmaceutical industry.^2^ The production of complex compounds from basic starting materials with high atom efficiency has been significantly enhanced during the past few decades through the development of transition metal-catalysed oxidative C–H functionalization.^3^ Various techniques have been investigated to selectively functionalize the C–H bond, and directing group-assisted C–H bond functionalization methods have received significant interest. This method creates a thermodynamically or kinetically preferred metallacycle intermediate by the transition metal coordinating to a heteroatom of the directing group. This approach has been used for both C(sp^2^)–H and C(sp^3^)–H bond functionalizations and has grown significantly over the past few decades.^4^ Compared to conventional cross-coupling processes, the direct conversion of C–H bonds into C–C or heteroatom bonds eliminates the need for the frequently complex pre-functionalization of starting materials and is a more cost-effective and ecologically friendly approach. Nevertheless, in benzene derivatives, additional regioselectivity-controlling components are needed because the difference in reactivity between the C–H bonds is typically less noticeable. In these situations, the preferred method for enabling site-selective functionalization is the application of directing groups (DG), such as amides, pyridines, or acetanilide. The DG brings the transition metal close to the C–H bond to activate, which raises the effective concentration of the catalyst at the site of interest and produces high levels of regioselectivity and improved reactivity.^5^ The traceless directing group, noncovalent contact, and steric effects of the catalyst–ligand–substrate all play guiding roles for the site selectivity; steric effects are no longer the only component influencing regiosectivity.^6^

Classically, directed C–H activation resulted in the introduction of groups in the *ortho* position of the benzene ring. This is because the transition-metal catalyst initially coordinates with the directing group, placing it close to the *ortho* C–H bond.^7^ In most of the C–H activation reactions, the metal catalyst plays a significant role in determining its mildness.^8^ Many catalytic systems were developed, but the majority of the conversions require harsh conditions and elevated reaction temperatures.^9^ In every given reaction, the C–H activation mechanism that works is mainly determined by the metal's identity and oxidation state. High-valent late transition metals, including Pd^II^, Rh^III^, Ir^III^, and Ru^II^ react by electrophilic routes, like coordinated metalation-deprotonation. Low-valent, electron-rich metals like Rh^I^ and Ir^I^ frequently form C–H bonds during oxidation. High valent metals typically catalyse C–H transitions without additional donor ligands. Electrophilic C–H activation frequently requires electron-poor catalysts, which contributes to understanding this phenomenon. Strong sigma-donor ligands can make the metal centre electron-rich and hinder C–H bond activation. Furthermore, DGs, typically associated with substrate molecules, perform as ligands on metal centres for reactivity and regioselectivity. High-valent catalysts have limited chances for fine-tuning their intrinsic features to improve reactivity under moderate reaction conditions. The scientific community has made significant advancements in understanding the mechanisms of C–H activation, paving the way for more complex catalytic systems.^8^

Among the metals, the second and third-row transition metals such as Pd, Rh, Ir, and Ru gained more attention for employment as catalysts for the direct C–H activation. Naturally, the earliest examples of moderate, low-temperature C–H conversions were performed using these metals.^8^ One of the most challenging goals was to alter the electronic properties of the catalyst and render it more electrophilic.^8^ This review will mainly focus on the C–H functionalization reactions catalyzed by palladium.^10^ Palladium was identified as one of the most prominent catalysts for C–H activation. Researchers in this field mainly focused on the discovery of new modes of catalysts and expanding its substrate scope. For the development of catalytic processes, Pd^0^/Pd^II^ and Pd^II^/Pd^0^ catalysis have both been widely used.^11^ The second-row transition metal Pd has an intermediate atomic size, which greatly adds to the broad range of reactivity and moderate stability of organopalladium compounds. The d-electron filled non-bonding orbitals allow Pd to readily participate in a wide range of coordinating reactions acting as Lewis base or nucleophilic species, and its valence-shell empty orbital at low energy allows it to behave as Lewis acids or electrophilic species.^12^ Pd^II^ catalysts are compatible with oxidants and can selectively functionalize cyclopalladated intermediates. These features make palladium one of the most attractive catalysts for the C–H activation transformation. Secondly, palladium is a versatile cyclometalater that stimulates C–H activation at both sp^2^ and sp^3^ sites, distinguishing it from other transition metals.^13^ Along with that, organopalladium complexes have non-polar C–Pd bonds due to palladium's moderate electronegative nature (2.2 on the Pauling scale), resulting in excellent chemo-selectivity and minimal reactivity with polar functional groups. These properties align with the ease with which palladium(0) and palladium(ii) can be interconverted under various conditions.^12^ The majority of the C–H activation reactions using palladium as a catalyst can be accomplished through ambient air and moisture, making them optimal for the application of organic synthesis.^10^ Observations of the enhanced reactivity of the electron-deficient palladium catalyst also led to innovative designs that reduce the energy barrier for the metal insertion. A remarkable advancement in the creation of novel catalysts has emerged from this discovery.^9^

Selective C–H activation and functionalization of heterocycles, including pyridine derivatives, poses unique challenges and potential. To allow the selective breaking of the C–H bond, a directing group (DG) that can effectively coordinate a transition metal catalyst (in this case, palladium) is necessary for the successful activation of the C–H bond on the *ortho* position. The nitrogen-based, electron-donating functional groups anilines and anilides have attracted a lot of attention as DGs in *ortho*-arylation techniques using Pd-catalyzed double C–H activation, frequently guaranteeing strong regioselectivities and good yields of cross-coupling products.^12^ The pyridine nitrogen site is an effective ligand that binds to a variety of transition metals. Coordination of the pyridine substrate can deactivate the metal centre by preventing coordination sites needed to activate C–H bonds. C–H activation approaches are most effective with pyridine derivatives, as *ortho* substitution reduces their coordinating ability. Nitrogen coordination is capable of directing the metal centre to certain C–H bonds in pyridine-containing substrates. This method involves forming five- or six-membered rings through directed C–H activation, which is common for heteroatom-containing directing groups other than pyridine.^14^ Chelation-assisted cleavage of C–H bonds at the pyridine-directing group is a highly effective method for functionalizing unreactive C–H bonds. In recent years, there has been significant progress in developing direct arylations of 2-arylpyridines that are more cost-effective.^15^ The nitrogen atom in the pyridine operates as a donor, resulting in the binding of the metal to generate (pyridine)N–metal bonds in many complexes. This is an essential factor for the ring to function as a directing group with the metal-based C–H activation. Using the metallacyclic system, the pyridine nucleus acted as a guiding group for the functionalization of the 2-aryl group with various functional groups, where the reduction of the metal was the key to the formation of new bonds.^16^ To enhance the pyridine derivative ring reactivity and regioselectivity, the electron-withdrawing groups (EWG) that are widely available, such as Cl, NO_2_, and CN, were used to carry out the reaction. The following works show the most efficient and convenient methodologies of C–H activation *via* palladium catalysts.^4^

## 2. C–H activation reactions of 2-phenyl pyridine using palladium catalyst

**Scheme 1:**
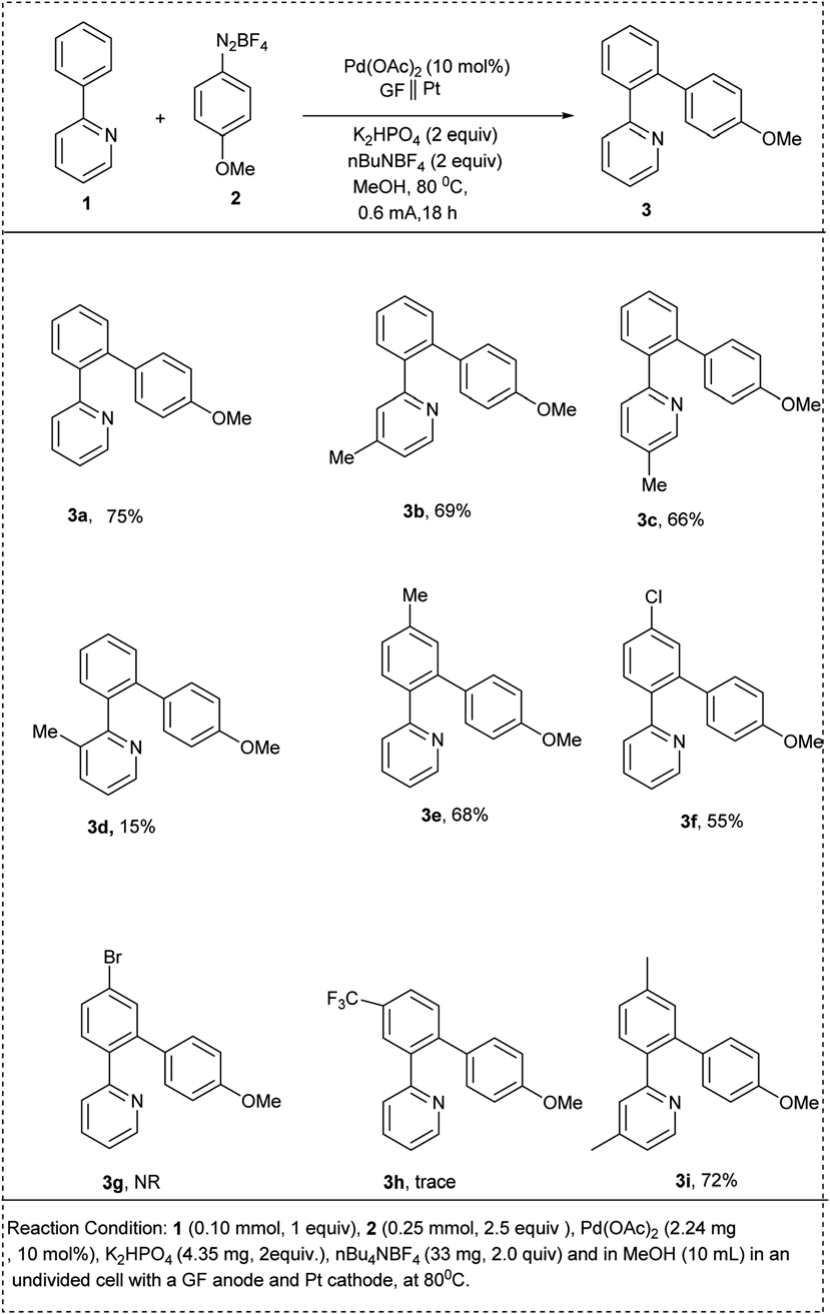
C–H activation of 2-phenylpyridine by electrochemical palladium-catalyzed *ortho*-arylation.

In 2025, Baroliya and his coworkers disclosed an electrochemical palladium-catalyzed *ortho*-arylation of 2-phenyl pyridine with various substituted arenediazonium salts under silver-free conditions. The studies showed that this reaction permits extremely selective arylation at the benzene rings *ortho* site with the aid of 2-pyridine auxiliary. The major advantages of this methodology include mild reaction conditions, good functional group tolerance, and broad substrate scope, which offers an efficient protocol for *ortho*-arylation C–H arylation. Notably, the utilization of electricity eliminates the need for hazardous or expensive chemical oxidants. This reaction utilized 2-phenylpyridine, 4-methoxyphenyldiazonium tetrafluoroborate salt and Pd(OAc)_2_ as an effective catalyst, K_2_HPO_4_, nBu_4_NF_4_ to afford the desired mono-arylated product with a significant yield of 75%. It was discovered that changing the current to a lower or higher value both resulted in reduced yields and that no appreciable quantity of arylated product was formed when the current was absent. Moreover, changing the electrode material significantly lowers the yield. Both electron-withdrawing and electron-donating functional groups performed well in this protocol, affording the desired products in excellent yields. The main characteristic of this reaction is the dual role of electricity in both catalytic reoxidation and reduction of arenediazonium ion and highly user-friendly undivided cell set-up (Scheme 1).^17^

**Scheme 2:**
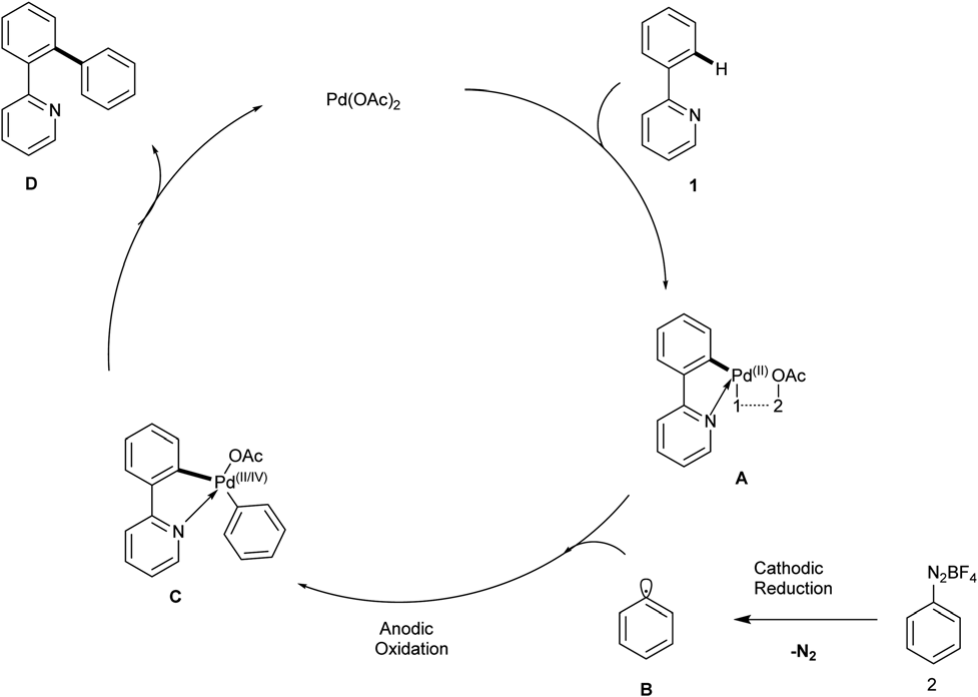
The plausible mechanism of the abovementioned reaction.

The proposed reaction mechanism (Scheme 2) follows a catalytic cycle in which a cyclopalladium species A was generated by pyridine-directed *ortho*-cyclopalladation. Further, this intermediate is cyclometalated with an aryl radical B, which was generated by the cathodic reduction of an aryldiazonium salt 2 and resulted in cyclopalladated intermediate C. Ultimately, Pd(i) or Pd(ii) and arylated phenylpyridine D are obtained by reductively eliminating intermediate C. Furthermore, palladium undergoes oxidation either during the coordination of the aryl radical B or following reductive elimination by anodic oxidation, which results in the regeneration of palladium(ii) to sustain the catalytic cycle.^17^

**Scheme 3:**
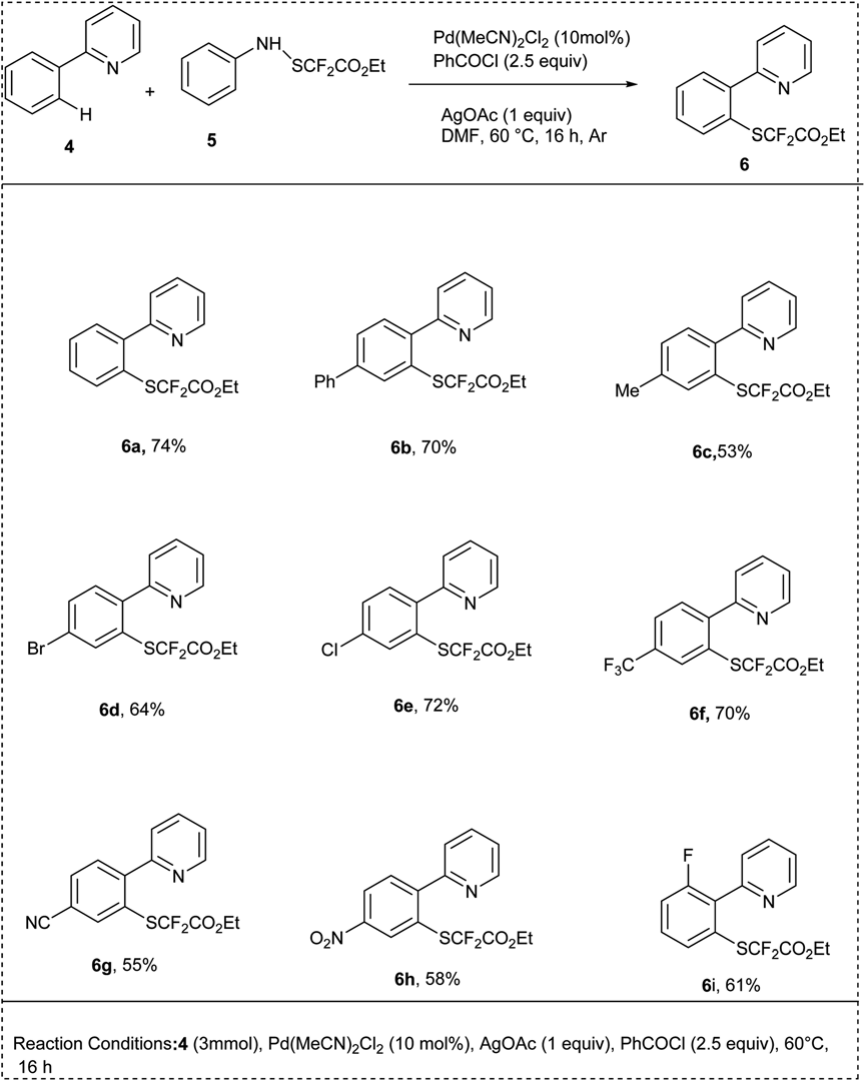
C–H activation of 2-phenyl pyridine using (ethoxycarbonyl)difluoromethyl thiolation reaction.

In 2022, Besset and his coworkers introduced an effective Pd-catalyzed (ethoxycarbonyl)difluoromethylthiolation reaction of C–H activation using (ethoxycarbonyl)difluoromethylsulfenamide reagent. The reaction was carried out by using 2-phenyl-pyridine and a (ethoxycarbonyl)difluoromethylsulfenamide reagent as a model substrate along with Pd(MeCN)_2_Cl_2_ as an efficient catalyst, DMF and additives AgOAc, PhCOCl used for activate DMF and the reagent to afford the desired product yield of 74%. This method was found to be useful when an optimal temperature was maintained, and the reaction efficiency was not improved by using higher reaction temperatures, whereas lower reaction temperature was detrimental to the reaction. The studies showed that the presence of the electron-donating substituents at the *para* position of the phenyl residue was well tolerated and resulted to afford good yields with substituents like methyl, phenyl, methoxy and an acetal group. However, the addition of electron-withdrawing groups such as trifluoromethyl, aldehyde, ester, and cyano did not impact the effectiveness of the reaction (Scheme 3).^18^

**Scheme 4:**
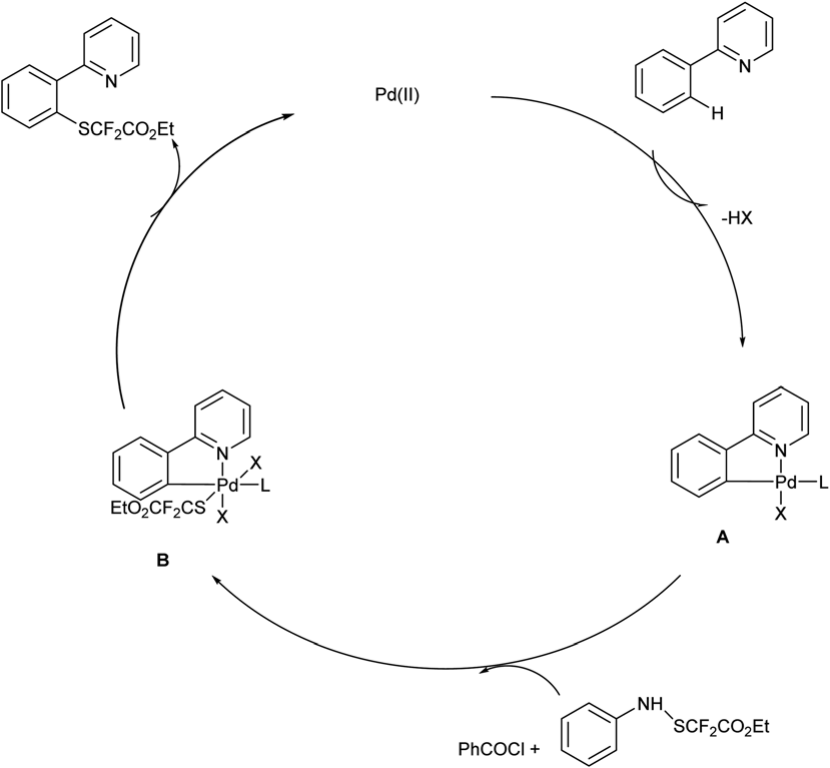
Plausible mechanism of the above reaction.

The proposed reaction mechanism (Scheme 4) follows a catalytic cycle in which the Pd(ii) catalyst would react with 2-phenylpyridine and form the palladacycle A. Further, the oxidative addition of reagent 1 activated with PhCOCl yields the high-valent Pd(iv) species B, and the reductive elimination generates the desired product and regenerates the Pd(ii) catalyst.^18^

**Scheme 5:**
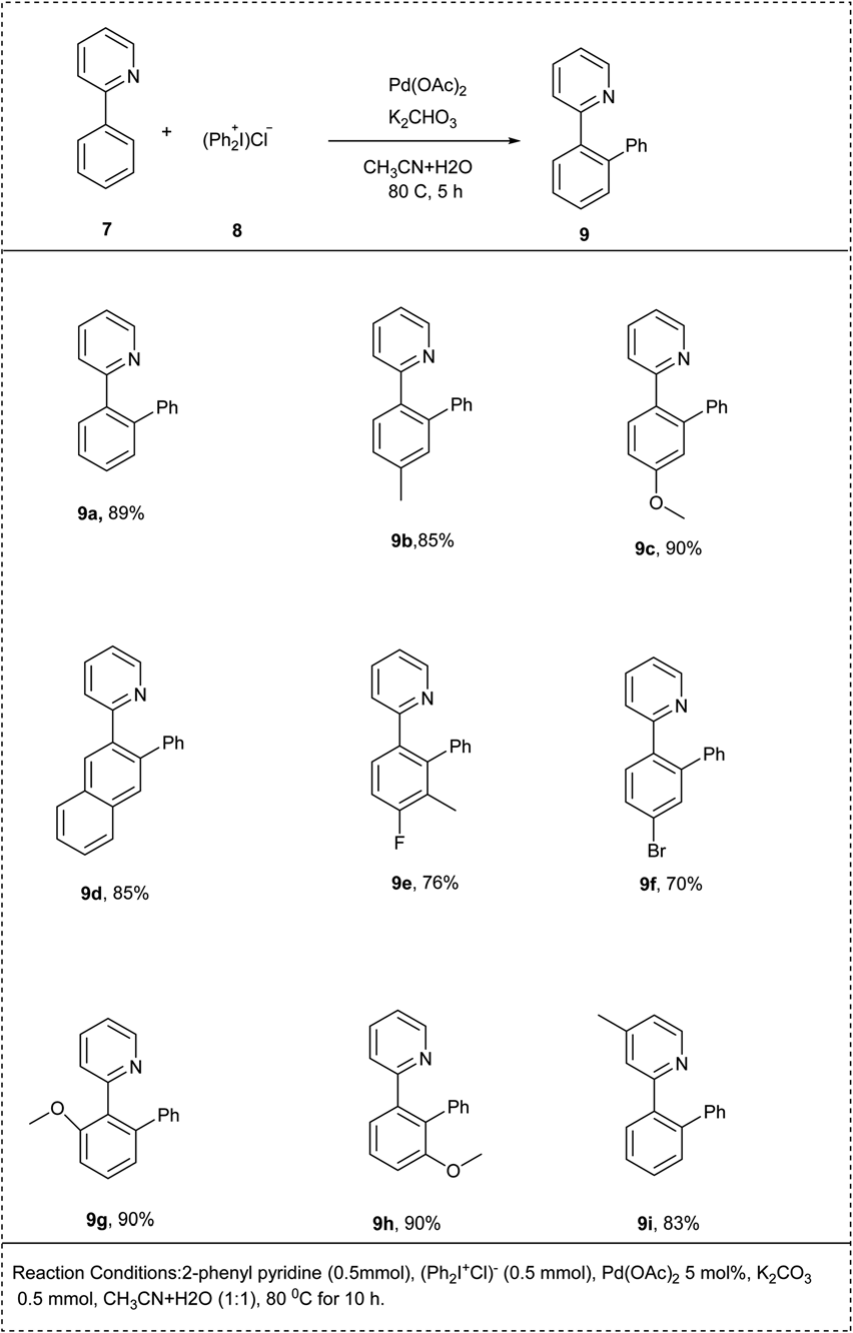
sp^2^ C–H activation of 2-phenylpyridine by *ortho* arylation with diphenyliodonium chloride.

In 2022, a one-pot, concise protocol for the high regioselective *ortho*-selective mono-arylation by C–H activation was developed by Raju and his coworkers. The reaction was carried out by using 2-phenyl pyridine and diphenyl iodonium chloride as an arylating reagent. The studies disclosed that in the presence of Pd(OAc)_2_, K_2_CO_3_ and solvent acetonitrile with water resulted to afford the desired product in a good yield of 89%. Notably, the substrate bearing electron-donating group on the aryl ring reacted smoothly to achieve an excellent yield of the desired product. Similarly, the bromo substituted 2-phenylpyridine also led to a good yield. Along with that, the 2-phenyl pyridine having *meta* substitution on the aryl ring gave the highly site selective products. This methodology contains a wide substrate scope and high functional group tolerance (Scheme 5).^19^

**Scheme 6:**
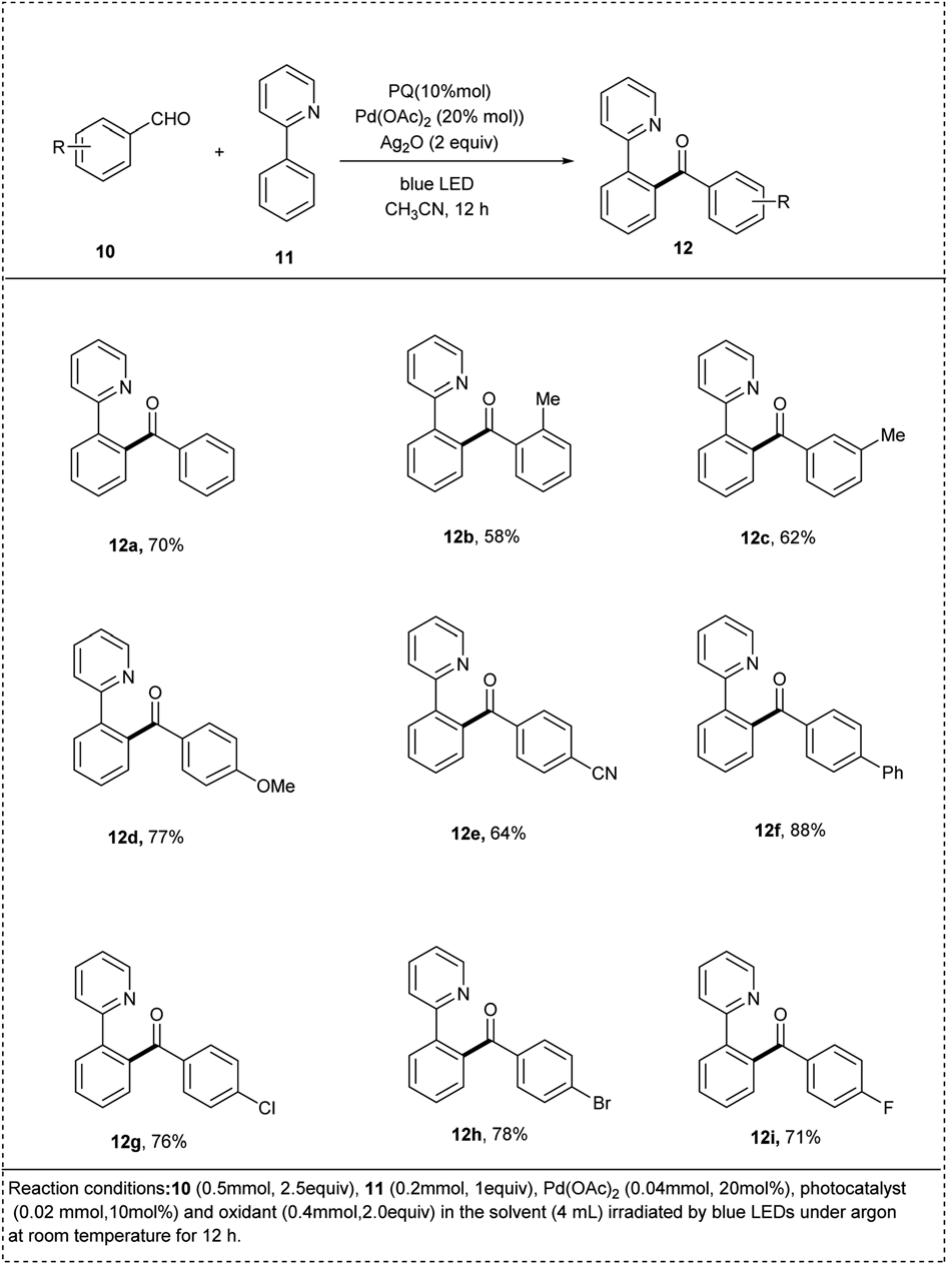
sp^2^ C–H activation of 2-phenyl pyridine by acylation of arenes.^3^

In 2021, an acylation of arenes with aldehydes through dual C–H activations was reported by Xia and his coworkers. The acylation was initiated by phenanthraquinone-catalyzed hydrogen atom transfer from aldehyde under visible light irradiation. This reaction utilised palladium acetate as the catalyst to achieve the desired product. This reaction focused on the photochemical acylation of 2 phenyl pyridine and found that Ag_2_O acts as an optimal oxidant to afford the desired product yield of 82%. The other oxidants such as [MnO_2_, Mn(OAc)_2_, O_2_, K_2_S_2_O_8_] were also utilized for the recycling photocatalyst and were found to give a poor yield due to the strong sensitivity of substrate towards these oxidants. The studies disclosed that both the electron-rich and electron-deficient substituents were well-compatible to achieve the desired product in excellent yields. The reaction was also conducted in the absence of a catalyst, which showed no desired product. The major advantage found was that the acylation was also done by both aromatic and aliphatic aldehydes and led to a moderate yield of the cross-coupling products (Scheme 6).^3^

**Scheme 7:**
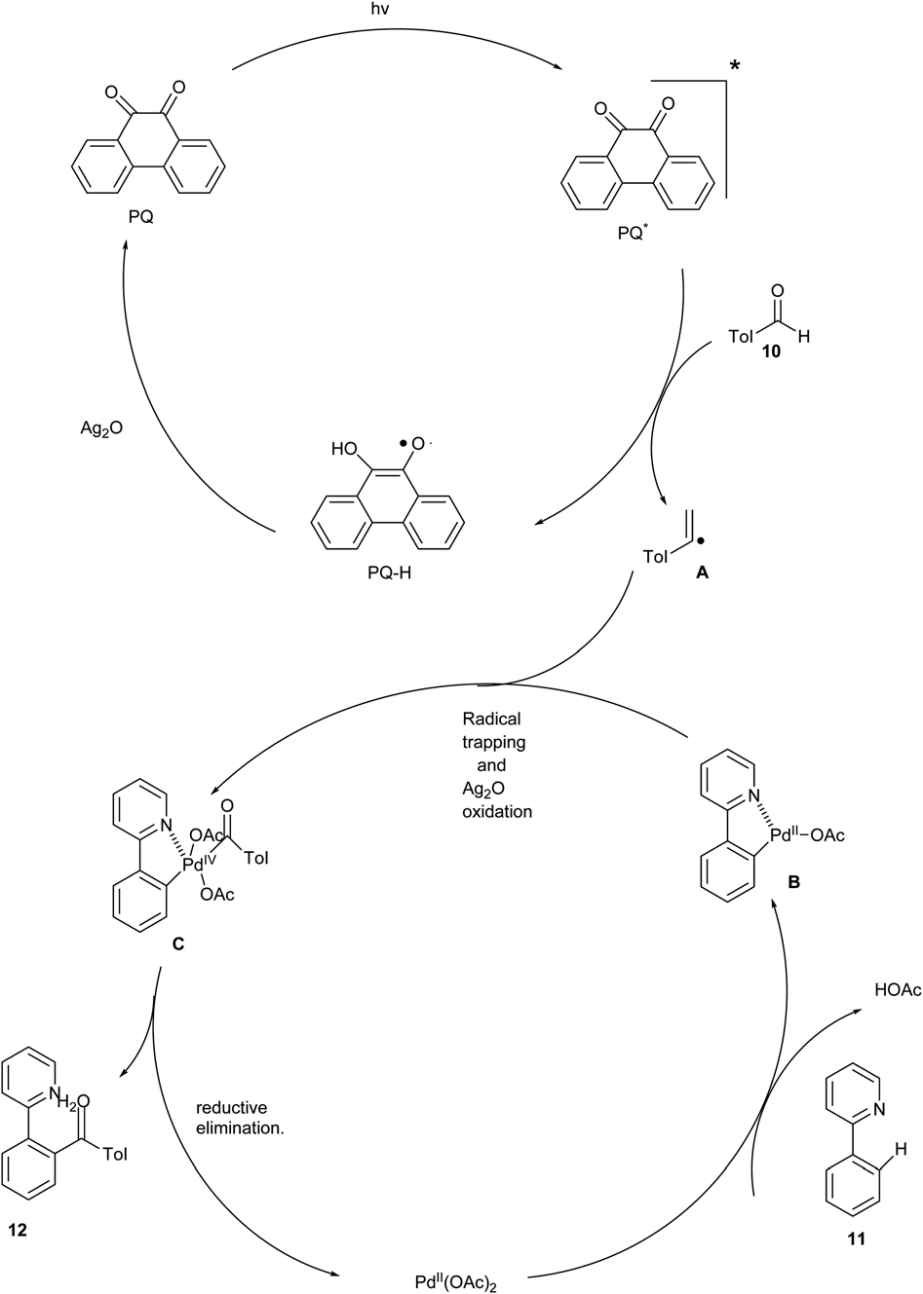
The plausible mechanism of the above reaction.

The proposed mechanism (Scheme 7) was initiated by the irradiation of the photocatalyst PQ to its electronically excited state PQ* by visible light. Then, hydrogen was abstracted from the aldehyde to form acyl radical A and PQ-H. Further, PQ-H was oxidised by Ag_2_O to recycle the photocatalyst. Meanwhile, a five-membered palladacycle intermediate B was generated by the reaction of 2-phenyl pyridine with Pd(OAc)_2_ through C–H activation. Additionally, Pd(iv) intermediate C was formed by the trapping of the photogenerated acyl radical by palladacycle B. Finally, the intermediate C undergo reductive elimination to form the coupling product 12 along with the regeneration of the Pd(ii) catalyst.^3^

**Scheme 8:**
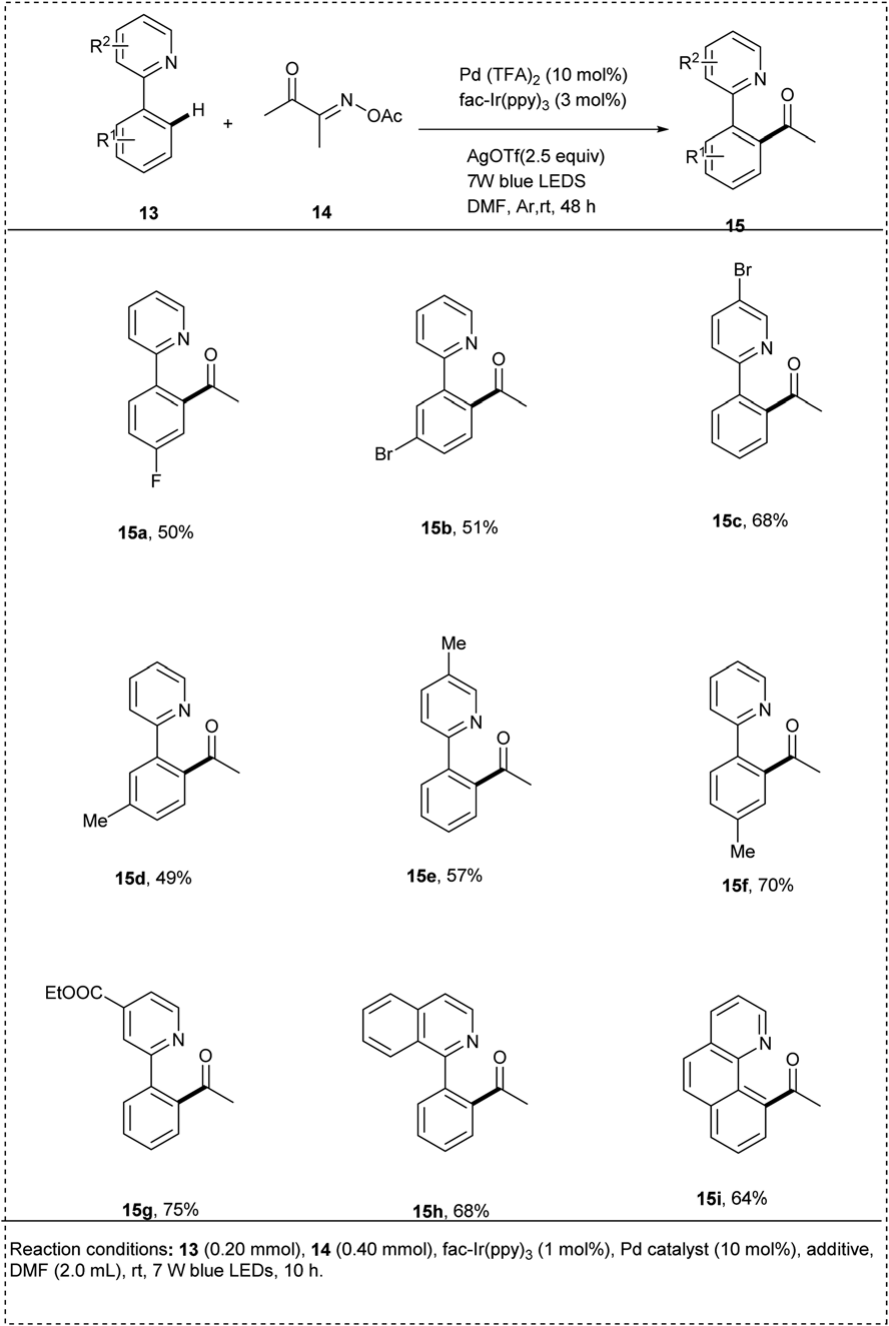
C–H activation of 2-phenyl pyridine by acylation with oxime esters.

In 2020, Liu described a novel method of utilizing oxime esters to direct *ortho* C–H acylation and iminyl radical-mediated dual photo redox/palladium-catalysed C–C bond cleavage of 2-arylpyridines. They initially utilized 2-phenyl pyridine and butane-2,3 dione *O*-acetyl oxime as model substrates. The reaction was carried out in the presence of the photocatalyst *fac*-Ir(ppy)_3_ and Pd(TFA)_2_ in DMF at room temperature under irradiation by 7 W blue LEDs. The desired reaction gave a yield of 21%. This method was found to be successful when the reaction time increased to 24 h, and the use of metal salts AgOTf as an additive significantly enhanced the reaction efficiency and afforded the desired products of yield 75%. In this methodology, no external oxidants and mild reaction conditions were used to carry out the reaction. The studies showed that the presence of a strong electron-withdrawing group in 2-phenylpyridine resulted in a decrease in yield. This redox-neutral protocol includes excellent regioselectivity and a high functional group tolerance (Scheme 8).^20^

**Scheme 9:**
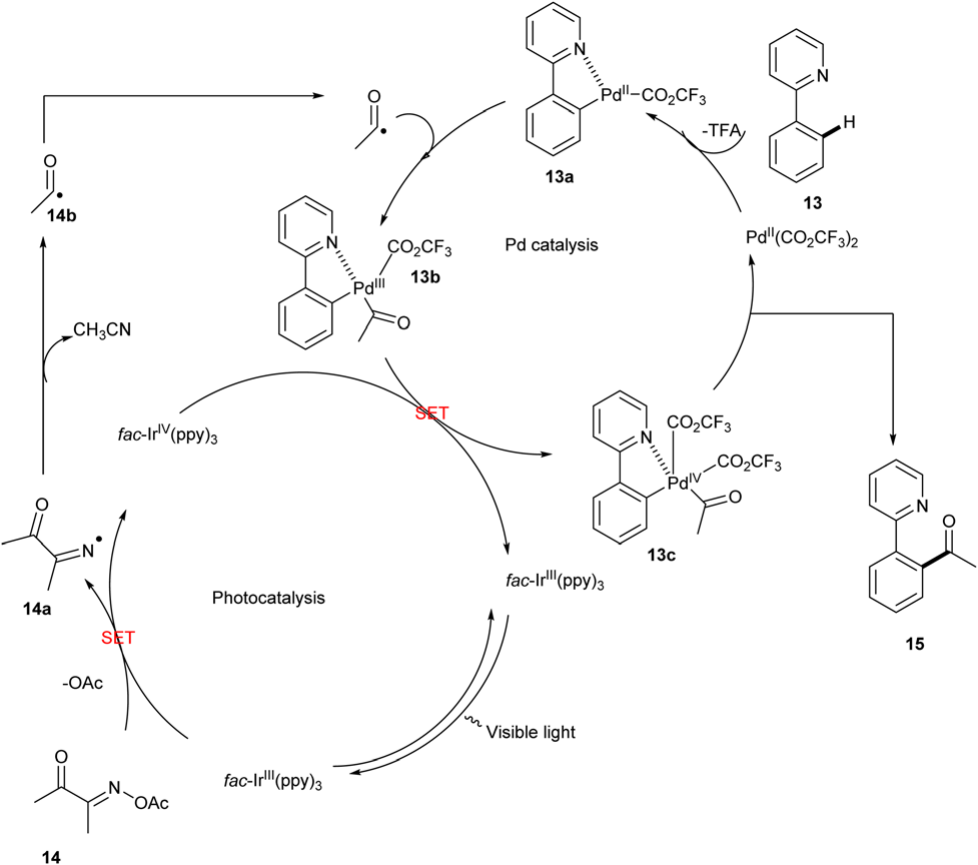
The plausible mechanism of the above reaction.

The proposed mechanism (Scheme 9) was generated by the irradiation of the photocatalyst *fac*-Ir^III^(ppy)_3_ and produces an excited *fac-Ir^III^(ppy)_3_, which undergoes a SET with oxime ester 14 to form iminyl radical 14a by C–O bond cleavage. More stable acyl radical 14b was generated by the further C–C cleavage of the iminyl radical. Meanwhile, 2-phenylpyridine 13 undergoes electrophilic palladation to form a five-membered cyclopalladated complex 13a, and it undergoes a reaction with acyl radical to form 13b, which then undergoes another SET oxidation with the oxidation state of *fac*-Ir^IV^(ppy)_3_ to form Pd^IV^ intermediate 13c and closes the photocatalytic cycle. Finally by the C–C bond formation of the 13C by reductive elimination results in the formation of the desired product 15.^20^

**Scheme 10:**
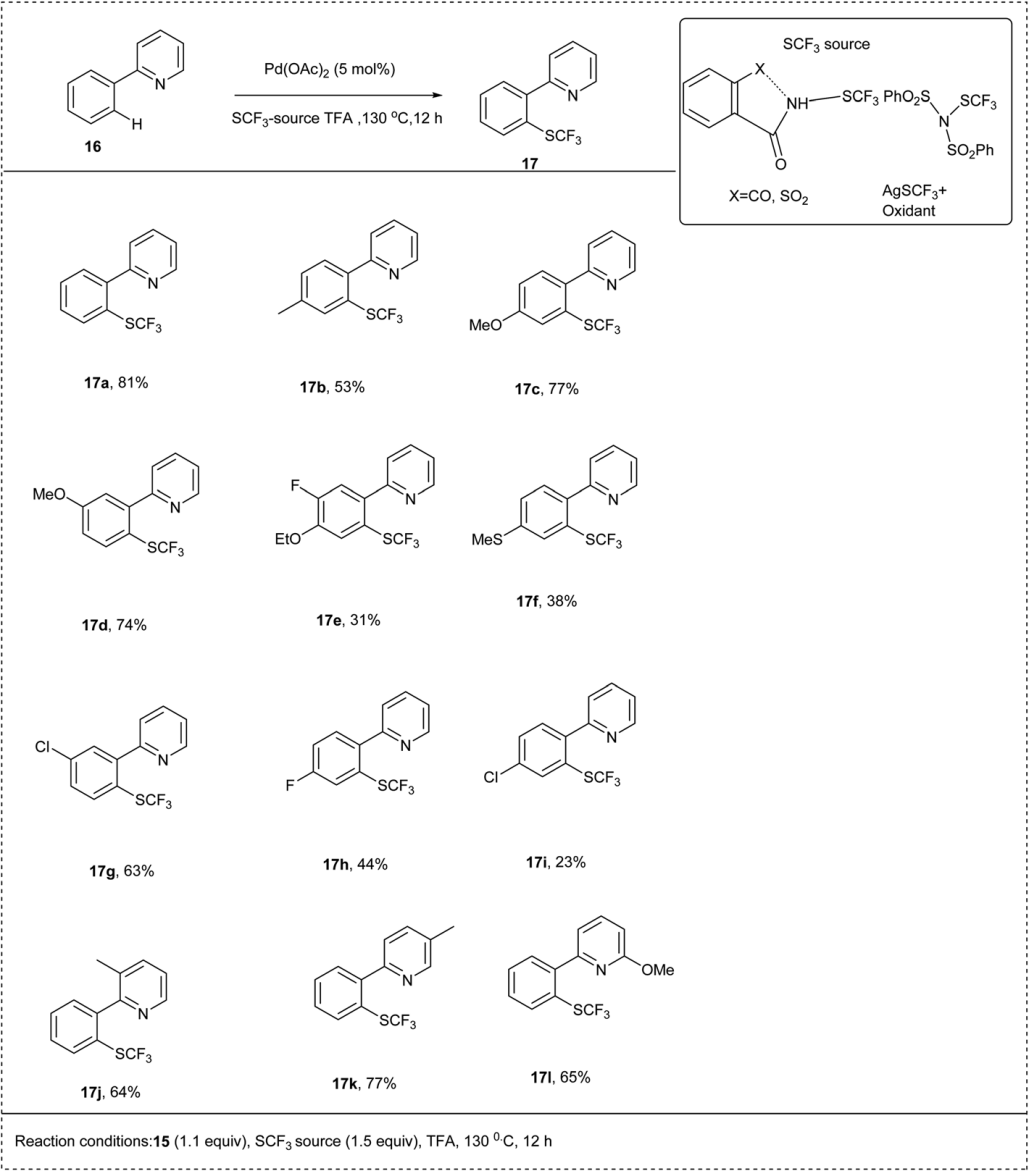
C–H activation of 2-phenyl pyridine by palladium-catalyzed trifluoromethyl thiolation.

In 2018, Anbarasan and his co-workers developed an efficient palladium-catalysed trifluoromethylationation of C–H activation by using a trifluoromethyl thiolating reagent. The reaction was carried out using 2-phenyl pyridine as a model substrate along with N-SCF_3_ reagents and palladium acetate catalyst in trifluoroacetic acid (TFA) and obtained the best yield of 90%. The electron-donating substituted aryl derivatives and electron-withdrawing halogen substituted groups reacted smoothly to achieve an excellent yield of trifluoromethyl thiolated product. This methodology contains a wide substrate scope and high functional group tolerance (Scheme 10).^21^

**Scheme 11:**
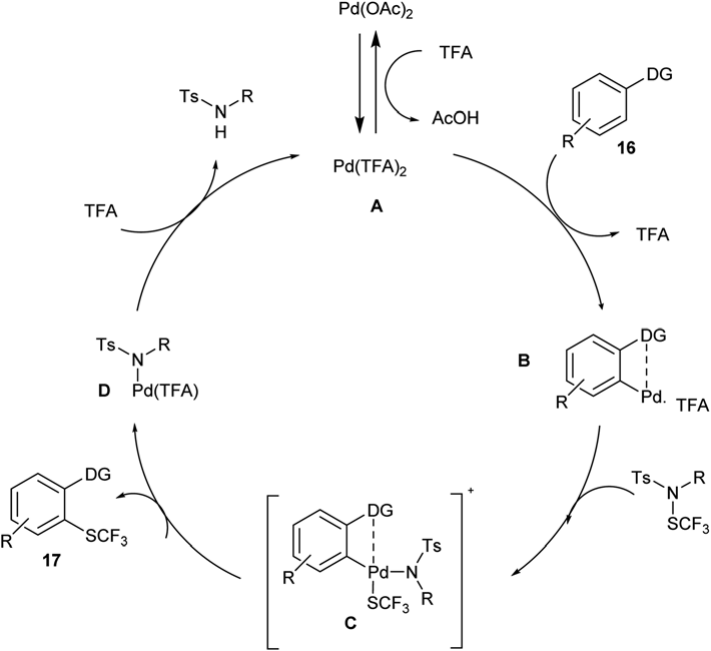
Plausible mechanism of the above reaction.

The proposed reaction mechanism (Scheme 11) follows a catalytic cycle in which an activated palladium species A was generated by reacting with palladium acetate and TFA; treating this with arene 16 would form a palladacycle B*via* chelation-assisted electrophilic metalation of an arene C–H bond. The oxidation-addition of N-SCF_3_ to B would form a Pd intermediate C, which through reductive elimination forms the product 17 and degenerates D. Further, the ligand exchange with TFA would complete the catalytic cycle and form an active A for the next cycle.^21^

**Scheme 12:**
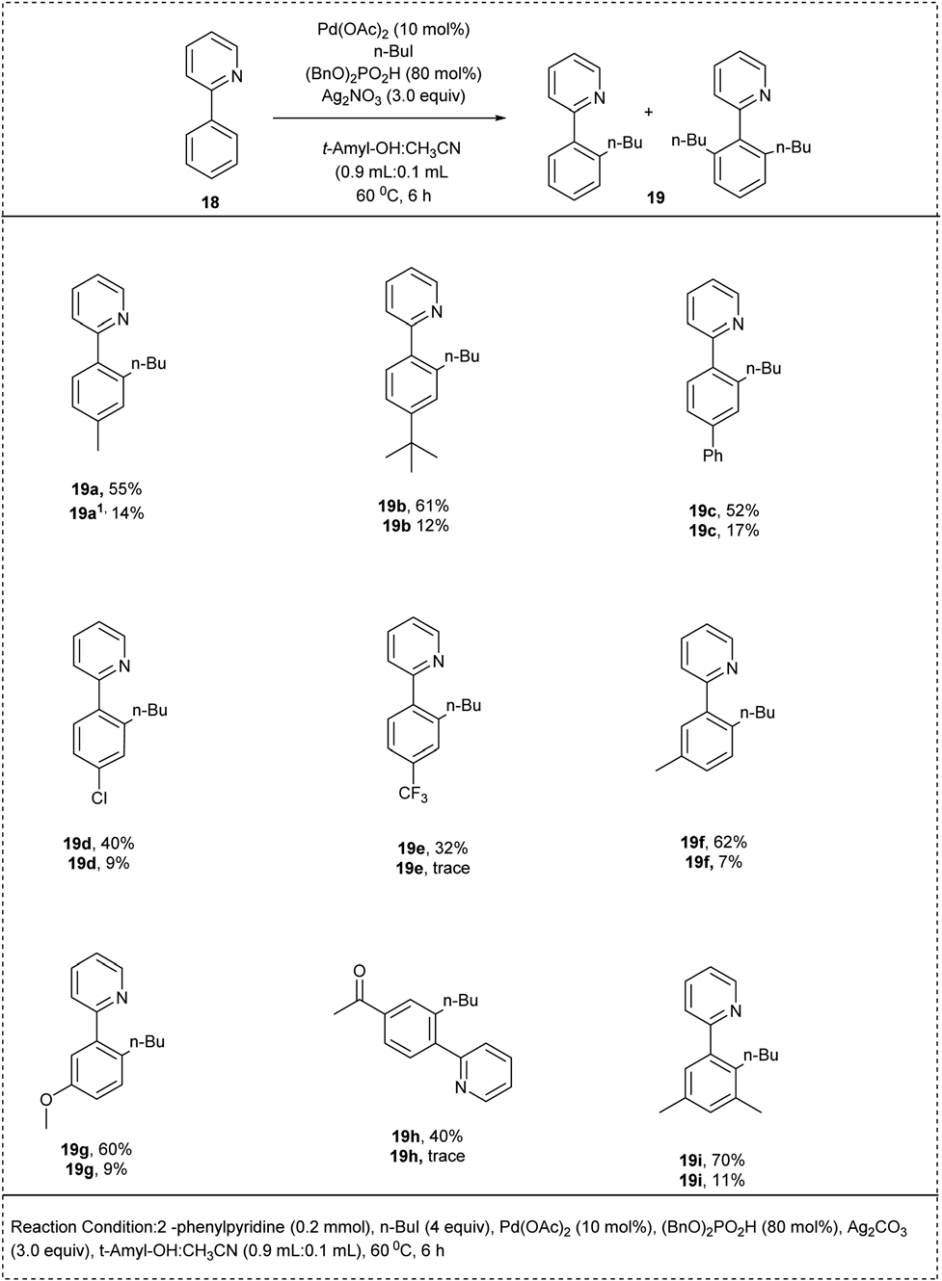
Pd catalyzed the C–H alkylation reaction of 2-phenylpyridine.

Zhang and his coworkers successfully developed a palladium-catalysed C–H alkylation reaction using 2-phenylpyridine and alkyliodine in 2017. The reaction utilized 2-phenylpyridine and alkyliodine as a model substrate along with Ag_2_CO_3_. The studies disclosed that using ligand (BnO)_2_PO_2_H and the solvent mixture of *t*-amylOH : CH_3_CN resulted in to affordment of monoalkylated products and dialkylated products with an excellent yield of 64% and 11%. Better yields of the alkylated products were found with *para*-substituted functional groups. Notably, the substrate bearing electron-withdrawing groups was found to be more reactive. However, the desired product yield was low compared to the electron-donating groups. Additionally, in the presence of a *meta*-methyl group, the reaction took place selectively at the less hindered position and produced a single monoalkylated product. The major advantage of this reaction was that it proceeded through a C, N-palladacycle obtained from 2-phenylpyridine as the key intermediate, and this key palladacycle intermediate exhibits great reactivity towards alkyl halides (Scheme 12).^22^

**Scheme 13:**
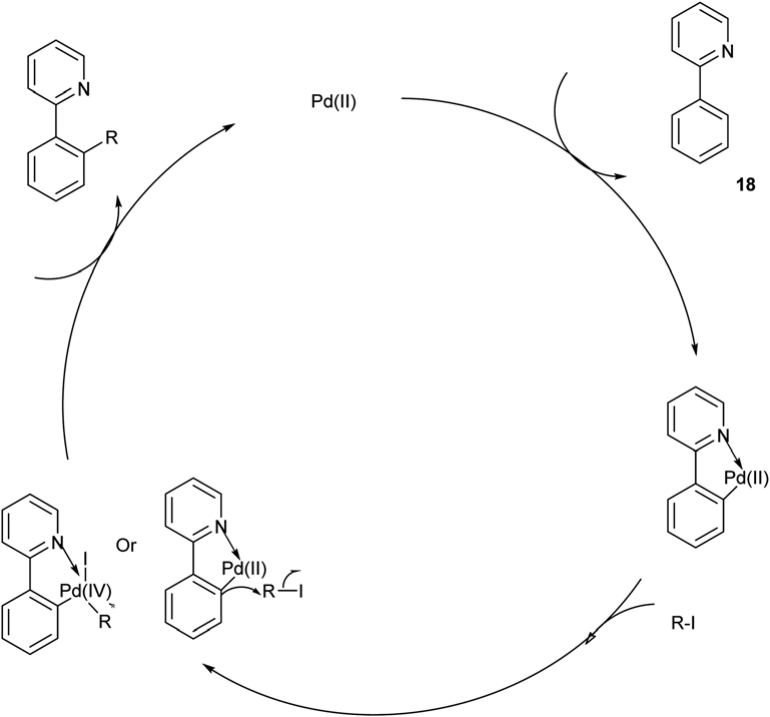
The plausible mechanism of the above-mentioned reaction.

The proposed reaction mechanism (Scheme 13) was initiated by the Pd^II^ catalyst's pyridine-directed C–H cleavage set off the catalytic cycle, creating the palladacycle. By reacting with alkyl iodide through either an oxidative addition or a substitution pathway, the resultant palladacycle produces the alkylated product and releases Pd^II^. Ag(i) ought to act as an iodide scavenger. To create the dialkylated product, the monoalkylated 2-phenylpyridine goes through the same catalytic cycle.^22^

**Scheme 14:**
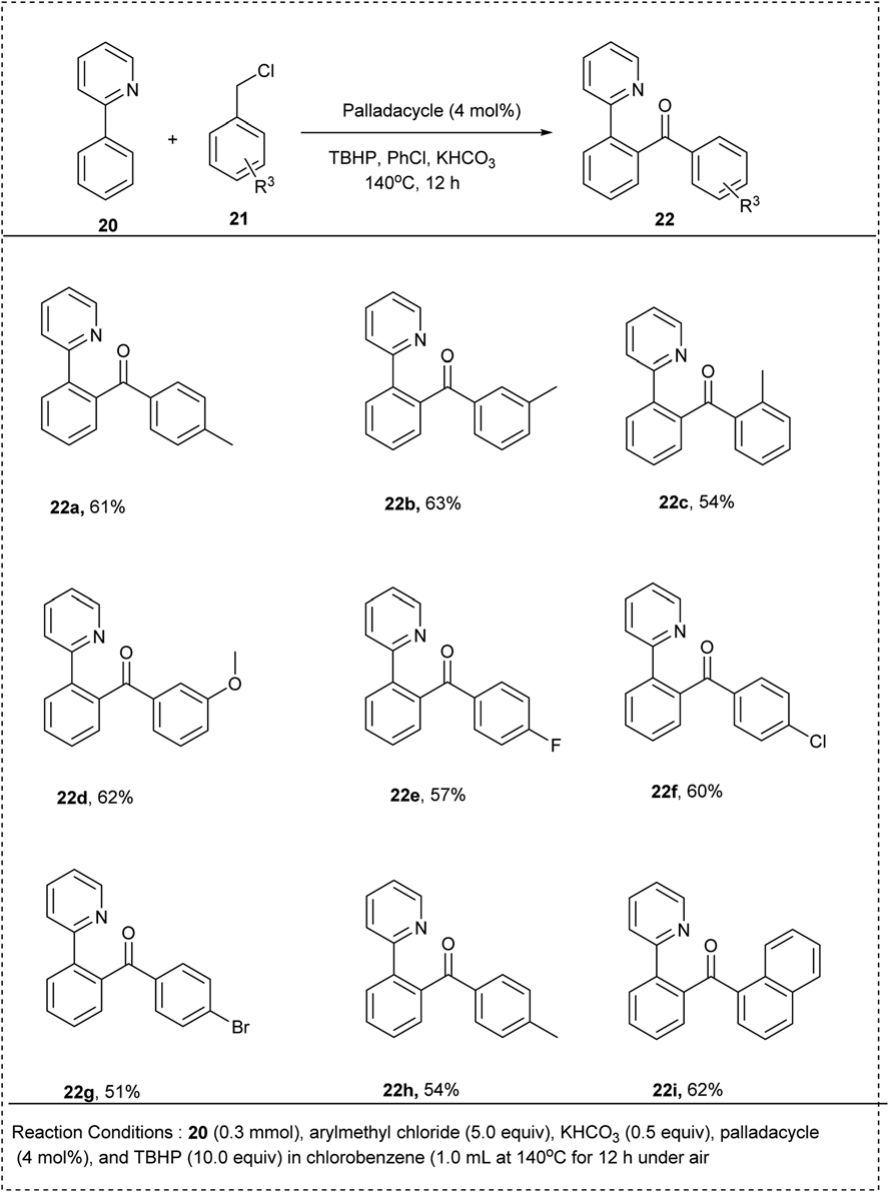
C–H activation of 2-phenyl pyridine with benzyl chloride by acylation.

In 2015, a novel method of ligand-directed *ortho*-CH chlorination and acylation with palladium-catalyzed and chemo-selective was successfully achieved by Yingjie Wu and his co-workers. The studies showed good regioselectivity for 2-aryl pyridines with a *meta*-substituent. This reaction was performed by using 2-phenylpyridine with benzyl chloride in the presence of *tert*-butyl hydroperoxide (TBHP) as the oxidant in chlorobenzene. The KHCO_3_ additive was found to be essential for this reaction, along with the palladacycle catalyst for achieving better yields of the expected products of high yield upto 75%. Notably, the substrate bearing moderate electron-withdrawing groups in the phenyl ring was able to generate the desired product with an excellent yield. The reaction is highly applicable, irrespective of the electronic nature of substrates. This method showed high regioselectivity for the substrates containing a *meta*-substituent in the phenyl ring (Scheme 14).^23^

**Scheme 15:**
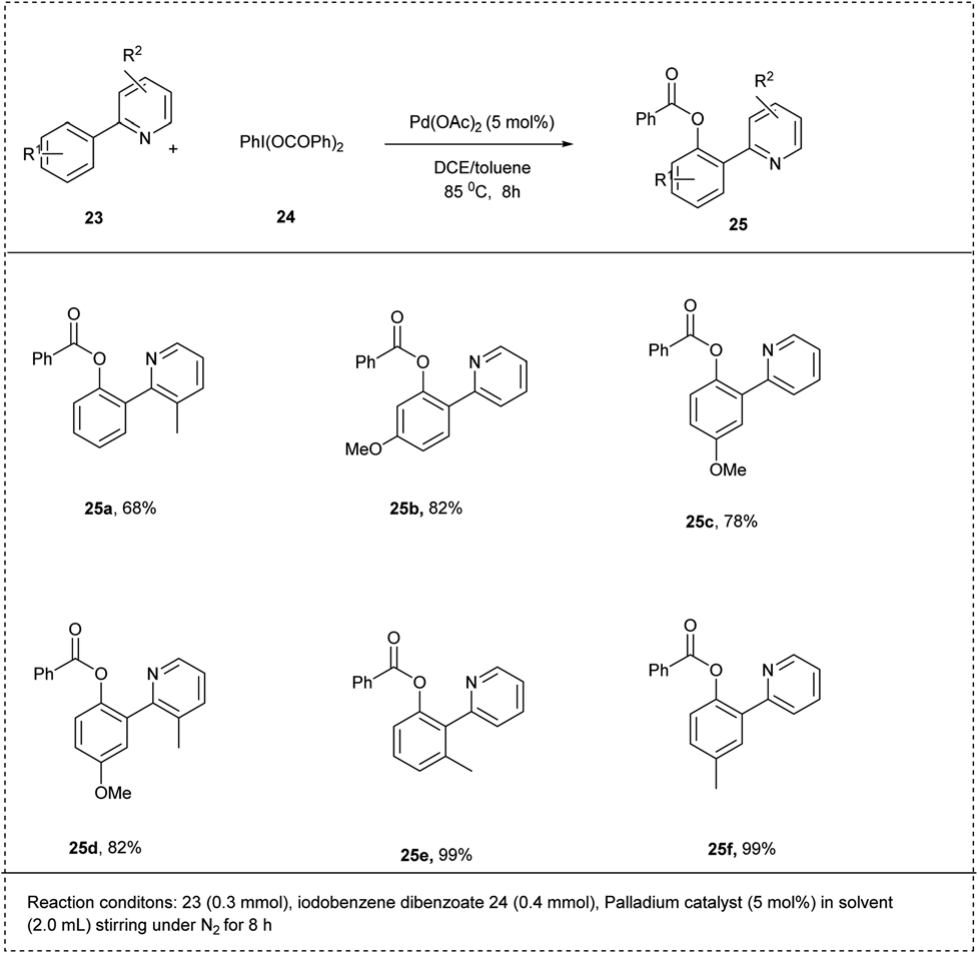
*Ortho* C–H benzoylation of 2-phenylpyridine using monobenzone dibenzoate.

In 2015, Qian Zhang and his coworkers elegantly developed a method of palladium-catalysed *ortho*-C–H-benzoylation of 2-arylpyridines using iodobenzene dibenzoates. The reaction was conducted using 2-phenyl pyridine and iodobenzene dibenzoate with Pd(OAc)_2_ as the catalyst and a mixed solvent of DCE/toluene, which greatly promoted the yield ratio of up to 85%. The studies showed that the product yield dramatically decreased under lower temperatures. A set of functional groups, such as Me, OMe, Cl, Br, F, and OCOMe, were fully compatible with this reaction. The 2-arylpyridines with electron-donating functional groups on the benzene ring reacted smoothly to achieve the required product in better yields than bearing electron-withdrawing groups. This method showed excellent regioselectivity when the benzene ring contained a *meta*-substituted group (Scheme 15).^24^

**Scheme 16:**
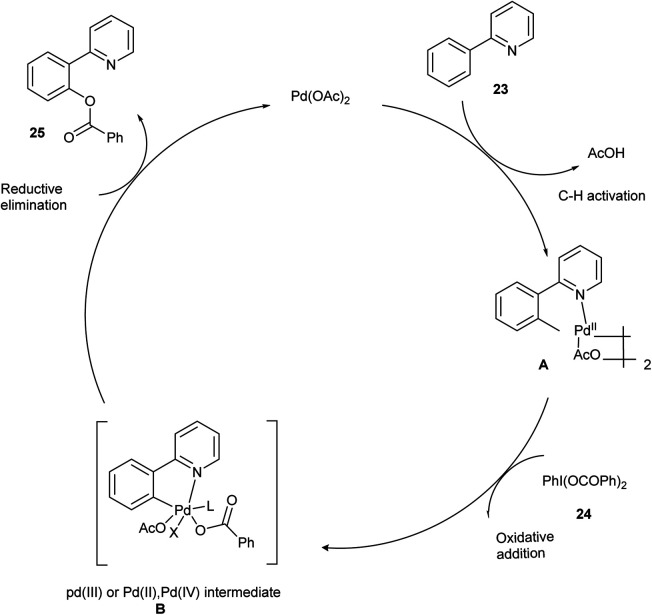
Plausible mechanism of the above-mentioned reaction.

The proposed mechanism (Scheme 16) begins with the formation of a bimetallic Pd(ii) complex A by the pyridyl-assisted *ortho* C–H activation cyclopalladation between 2-phenyl pyridine 23 and Pd(OAc)_2_. Then, the reaction between the complex A and 24 resulted in the formation of oxidative addition intermediate B, and the reductive elimination of intermediate B led to the desired product 25.^24^

**Scheme 17:**
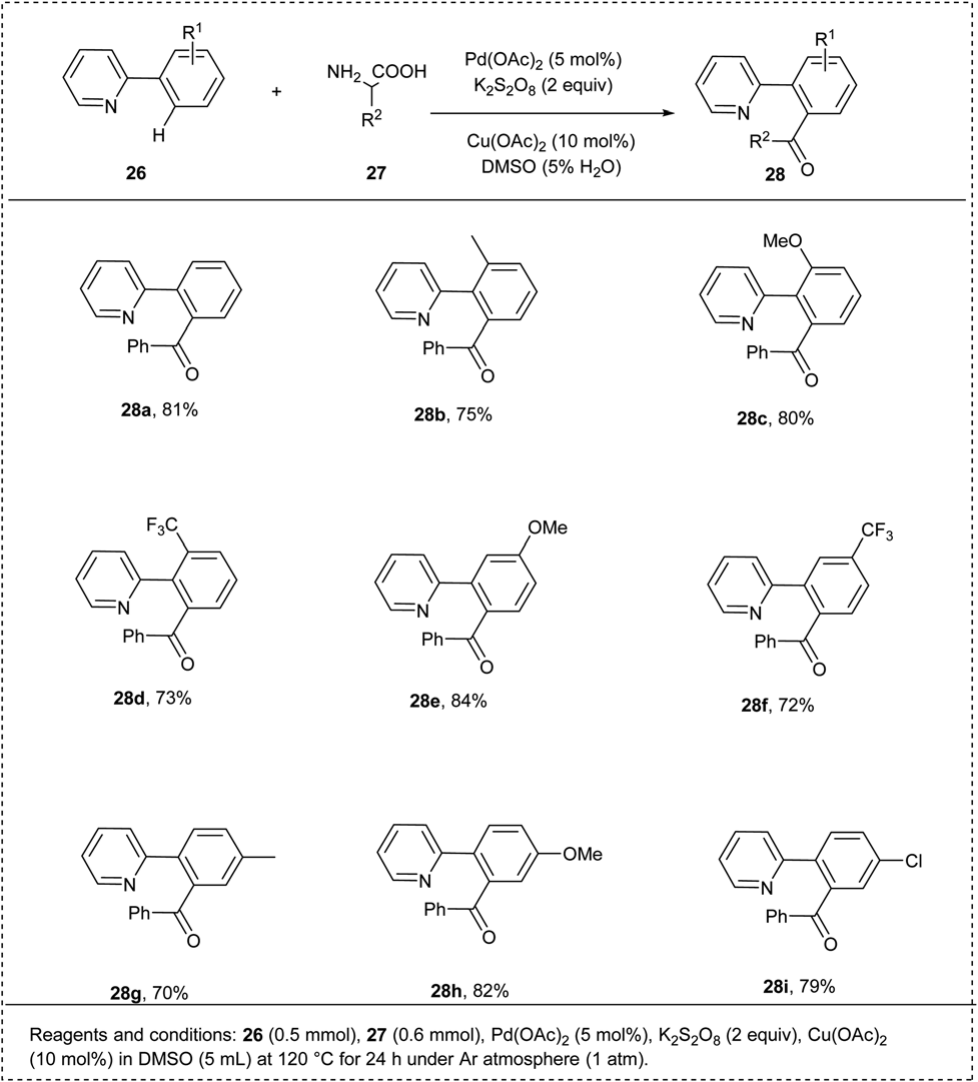
C–H activation of 2-phenyl pyridine by acylation with arylglycine derivatives.

In 2015, Feng and coworkers disclosed a palladium(ii)-catalysed *ortho* acylation of a wide variety of arenes using arylglycine through C–H activation. They initiated the reaction with 2-phenyl pyridine and phenyl glycine as a model substrate. The studies disclosed that when the reaction was conducted with Pd(OAc)_2_ and K_2_S_2_O_8_ in DMSO (including H_2_O), the desired product was formed in 52% yield. A slightly higher yield was observed when Cu(OAc)_2_ was introduced into the reaction. The electron-deficient substituents show higher activity and afford the desired products in good yields. The major advantages of this methodology include mild reaction conditions, good functional group tolerance, and broad substrate scope (Scheme 17).^25^

**Scheme 18:**
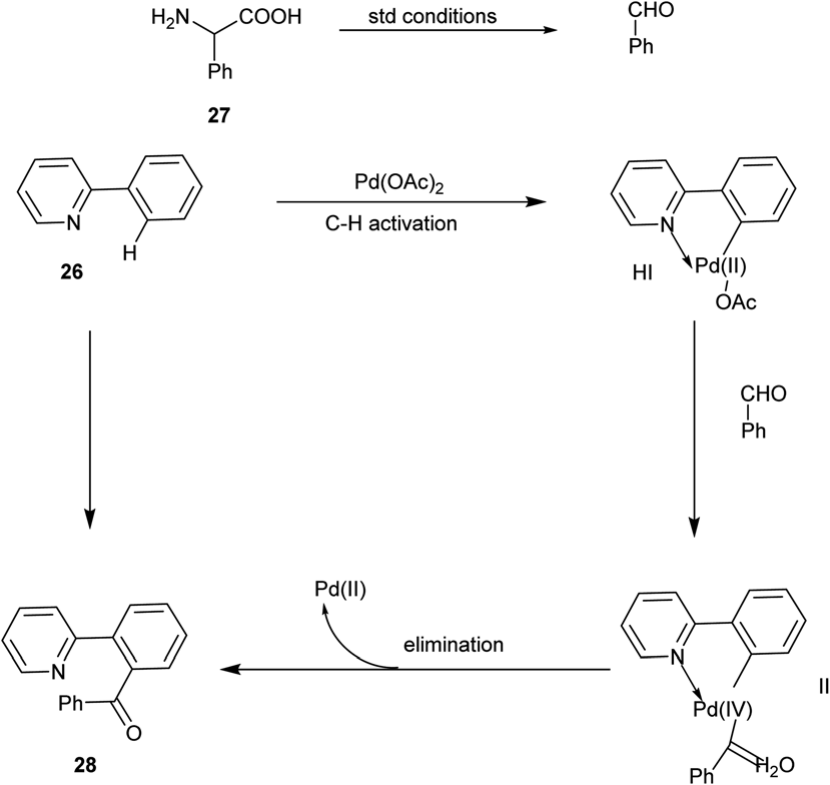
The plausible mechanism of the above reaction.

The proposed reaction mechanism (Scheme 18) was initiated by the reaction of an active palladium catalyst with 27 by chelation-directed C–H activation and generates intermediate I. Further, 26 gets oxidized to PhCHO, and by the oxidation addition, it leads to the formation of the intermediate II. Finally, the desired product 28 was formed by an elimination process, and active palladium(ii) was again generated for the further catalytic cycle.^25^

**Scheme 19:**
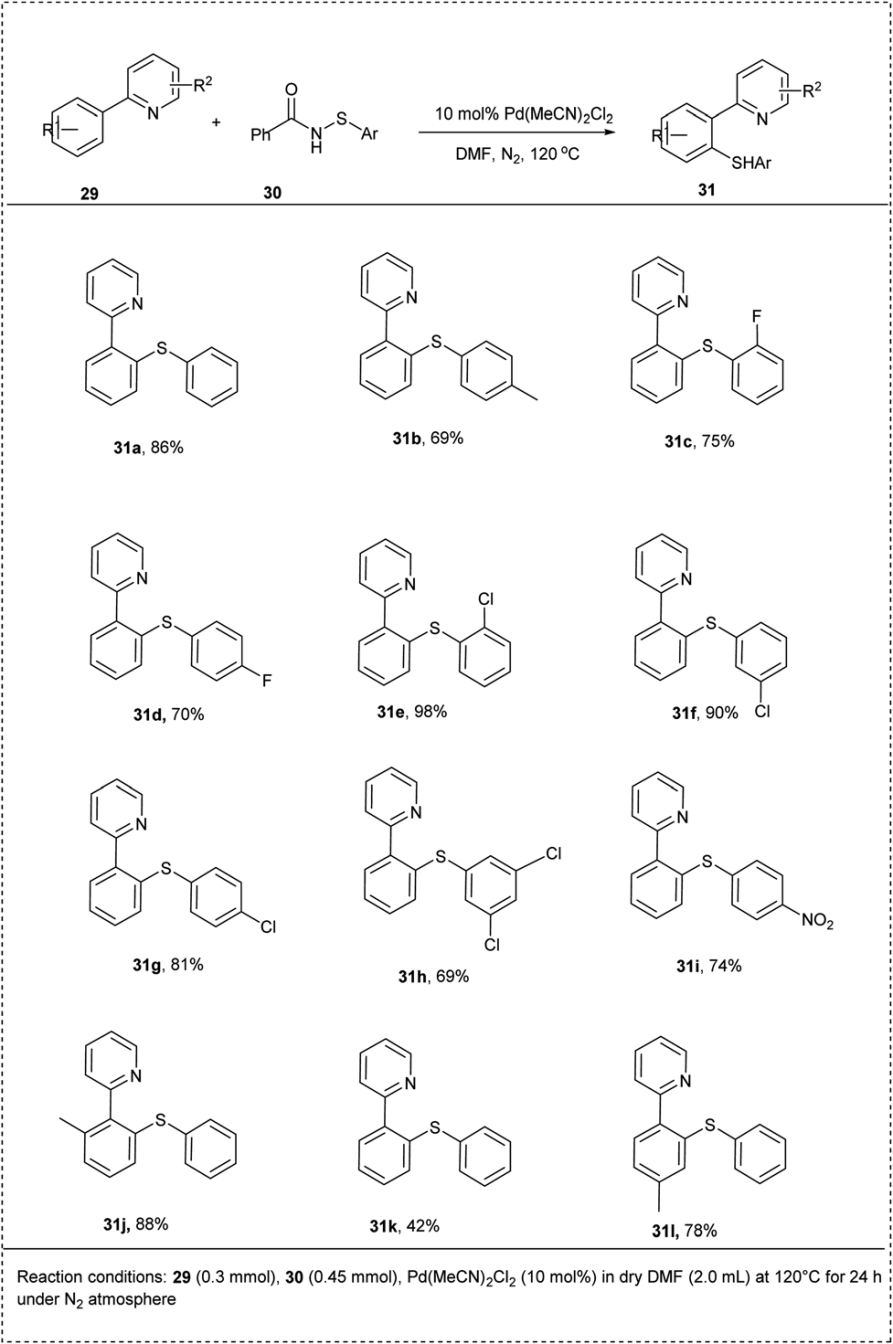
C–H activation by *ortho* mono-thiolation of arenes.

In 2015, Xing-Song Zhang and co-workers performed direct sulfenylation of arenes with *N*-arylthiobenzamides through a palladium-catalysed protocol under mild reaction conditions by using *N*-arylthioenzamides itself as a suitable oxidant. This reaction utilized 2-phenylpyridine and *N*-(phenylthio)benzamide as starting materials and Pd(MeCN)_2_Cl_2_ as a catalyst, along with DMF, to afford excellent yields of the expected products. Both electron-donating and electron-withdrawing substituted on aryl thiol moiety were well tolerated in this reaction. Moreover, this reaction worked smoothly when the amount of substrate was increased and resulted in an 86% yield. Substrates with strong electron-deficient groups showed the desired product in good yield. Arenes containing a bulky directing group lead to thiolation products in excellent yields. The major characteristics of this method include excellent functional group tolerance and avoiding the addition of multi-equivalent oxidants, which provides a new strategy for the green and atom-economical synthesis of aryl sulfides (Scheme 19).^26^

**Scheme 20:**
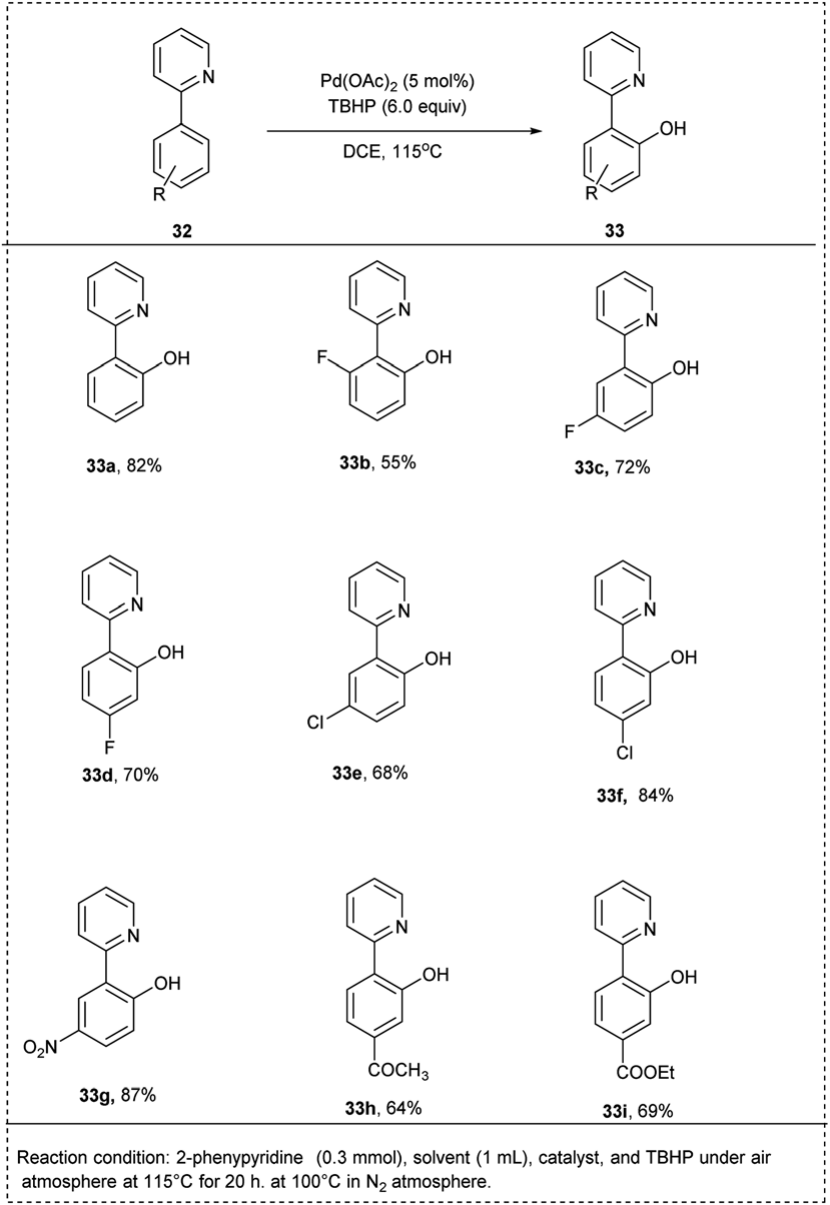
C–H activation by hydroxylation of 2-phenyl pyridine using TBHP as oxidant.

In 2015, Sun and co-workers introduced a pyridyl-directed homogeneous hydroxylation of aryl C–H bond using Pd-catalyzed for efficient synthesis of phenols. The reaction was performed with 2-phenyl pyridine as a reactant, and *tert*-butyl hydroperoxide (TBHP) was used as a sole oxidant. The studies showed that this oxidation reaction could be catalyzed by Pd(OAc)_2_ to give the hydroxylation product 2-(pyridine-2-yl)phenol in 58% yield. In DCE, the reaction proceeded smoothly and gave the highest yield. The reaction temperature was found to be crucial to attain excellent yield. The studies disclosed that the *meta* or *para*-halogenated substrates were well tolerated with the process and afforded the desired hydroxylated products with an excellent yield. The 2-arylpyridines, which possessed electron-deficient functional groups on the benzene ring, reacted smoothly to achieve the required product in better yields than bearing electron-rich functional groups. This methodology was found to be favourable for *meta*-substituted substrates proceeding hydroxylation at the less steric hindered position with high regioselectivity and had significant yield (Scheme 20).^27^

**Scheme 21:**
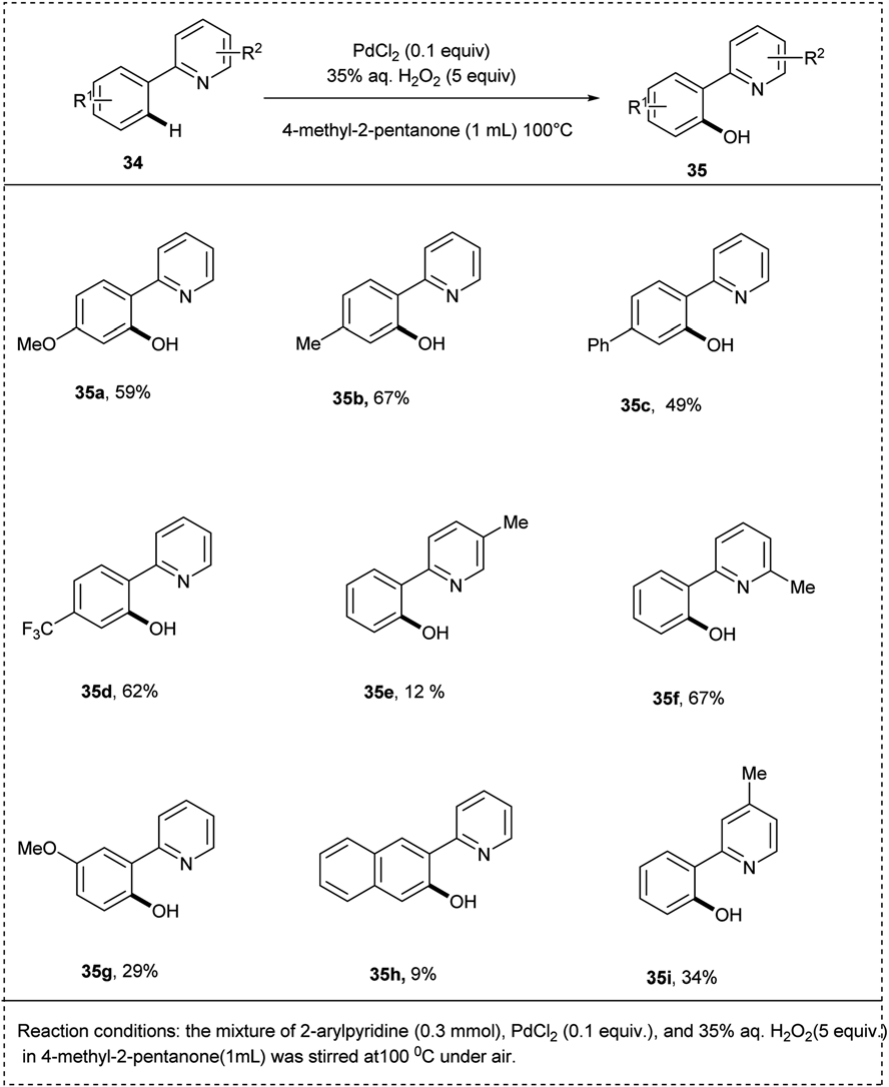
C–H activation by direct *ortho*-hydroxylation of 2-phenyl pyridine using palladium chloride.

Ithoih and coworkers successfully developed the direct *ortho*-hydroxylation of the 2-phenyl pyridines using palladium(ii) chloride and aqueous hydrogen-peroxide *via* C–H activation in 2015. This reaction includes PdCl_2_ as a catalyst along with high atom efficiency hydrogen peroxide, resulting in a yield of 75%. Both electron-withdrawing and electron-donating functional groups performed well in this protocol, affording the desired products in excellent yields. Studies disclosed that the *meta*–*ortho*-substituted compounds were converted to the corresponding phenols in low yields (Scheme 21).^28^

**Scheme 22:**
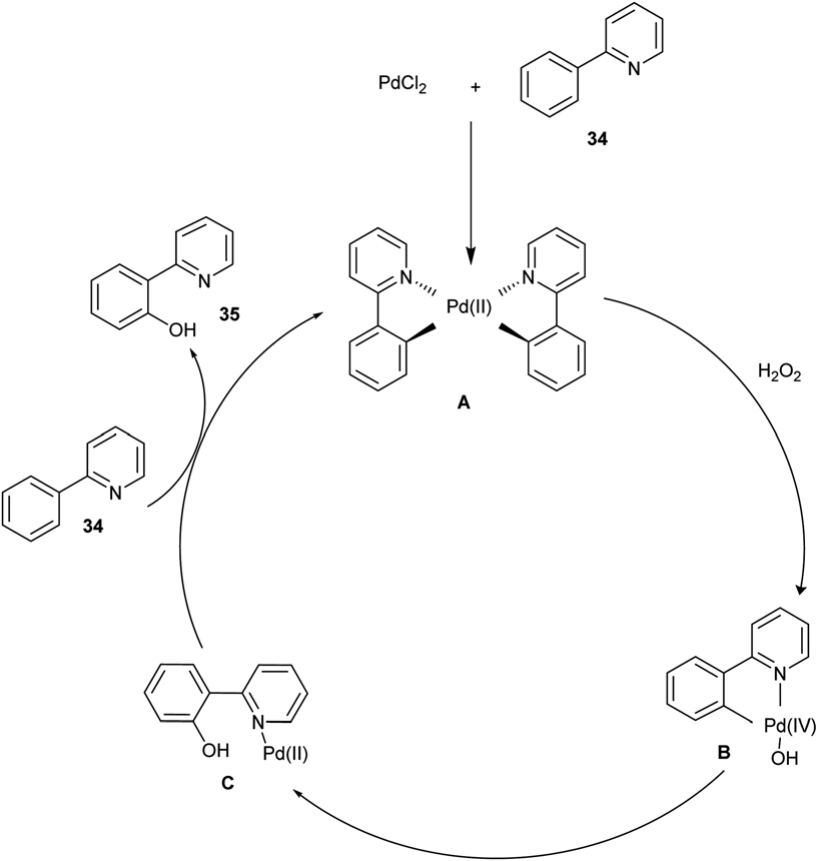
The plausible mechanism of the above reaction.

The mechanism involved here (Scheme 22) is, initially, the catalyst PdCl_2_ and substrate 34 reacted to form palladacycle A and was oxidized with hydrogen peroxide to form a high-valent Pd complex B. After that, by reductive elimination, B was converted to C. A hydroxylated product 35 was released from C*via* ligand exchange, and A was regenerated for the further catalytic cycle.^28^

**Scheme 23:**
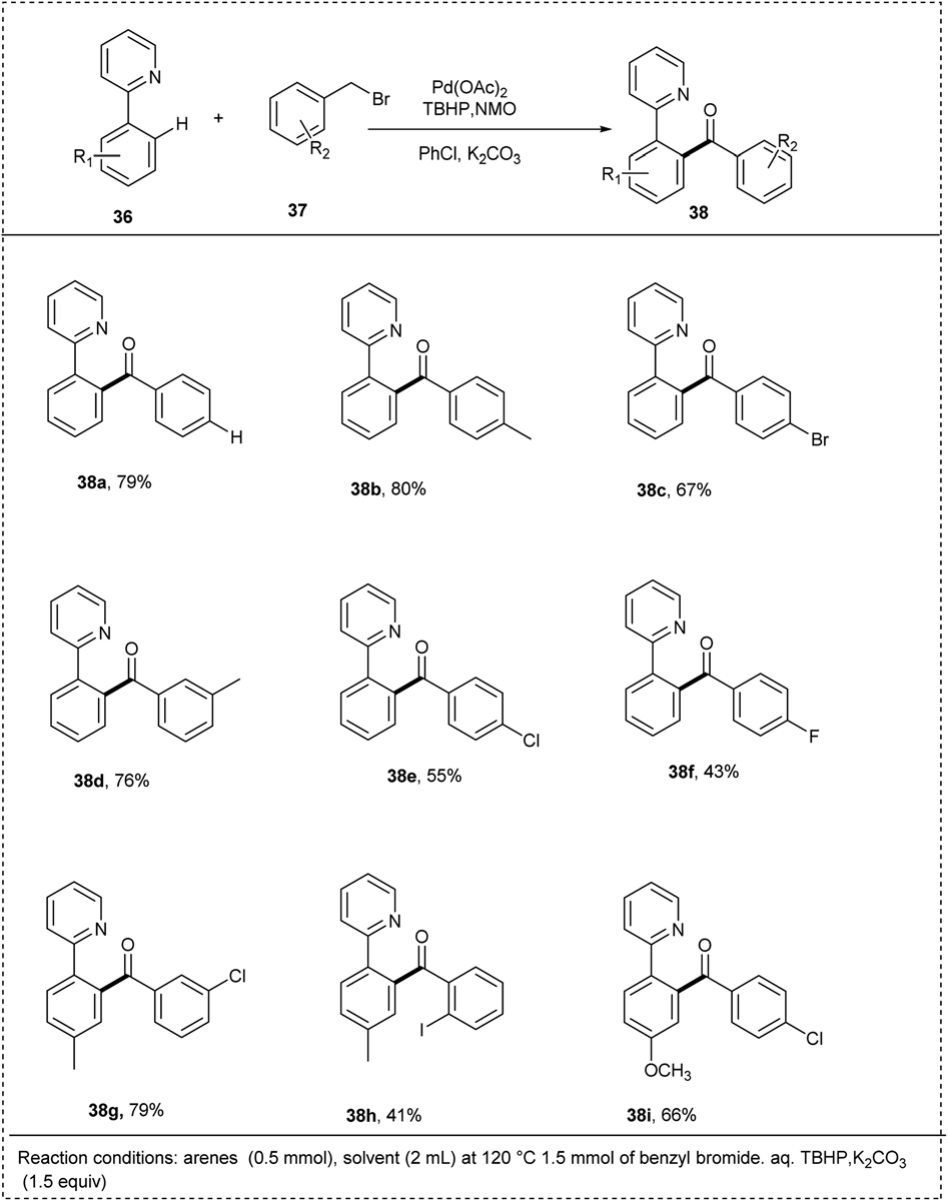
C–H activation of 2-phenylpyridine by *o*-arylation with benzyl bromide.

In 2015, Patel and coworkers elegantly developed a method for the effective *ortho*-arylation of 2-phenyl pyridine through palladium-catalyzed C–H activation in the presence of benzyl bromide. The initial reaction was conducted by 2-phenyl pyridine and benzyl bromide as model substrates with a Pd(OAc)_2_ as the catalyzed and oxidant TBHP and oxidant *N*-methyl morpholine *N*-oxide (NMO) in chlorobenzene, which greatly promoted the yield up to 79%. The studies disclosed that this reaction was carried out under a mild condition k_2_CO_3_ and made a significant impact on the yield of desired products. Electron-rich functional groups on the substrate reacted smoothly to afford desired products in better yields due to the better chelation of the palladium catalyst. The studies showed that both the activating and deactivating groups on the benzyl bromide efficiently reacted with 2-phenyl pyridine, and the desired product was obtained (Scheme 23).^29^

**Scheme 24:**
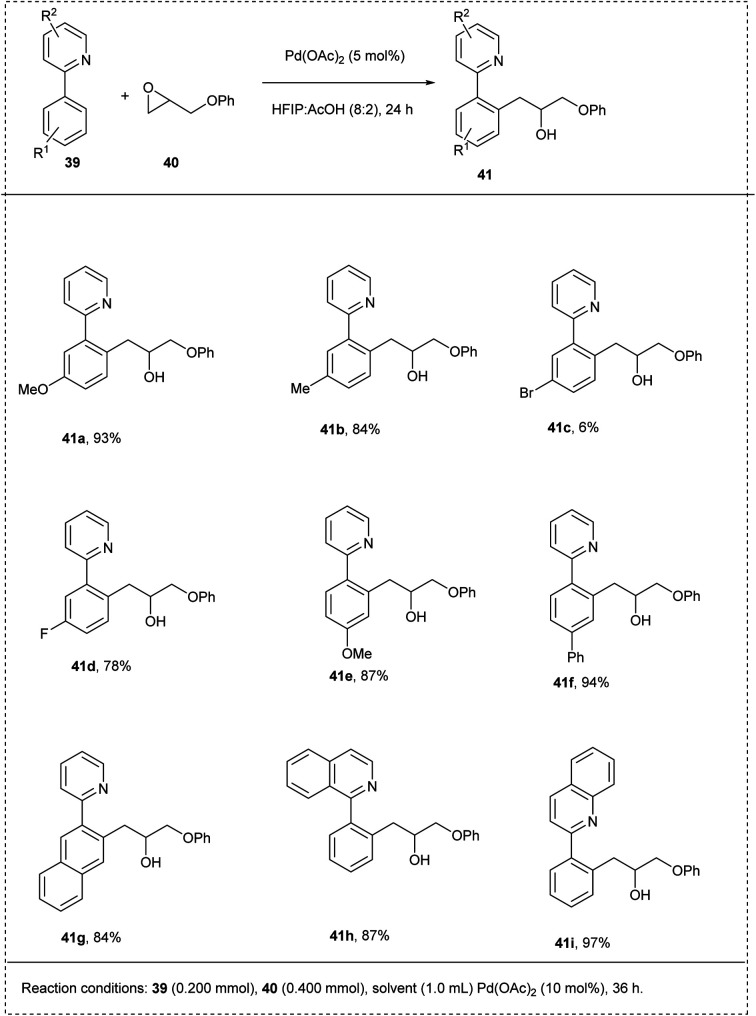
C–H activation of 2-phenylpyridine using oxirane.

In 2015, Wang and his co-workers synthesized a coupling reaction between arenes with pyridyl using palladium-catalyzed C–H activation. The reaction was carried out using 2-phenyl pyridine and 2-(phenoxymethyl)oxirane as model substrates using Pd(OAc)_2_ as a catalyst, obtaining a good yield of 92%. The major characteristics of this methodology include excellent functional group tolerance carried out at room temperature without any additives and obtained excellent yield on a gram scale itself. The studies disclosed that both electron-rich groups and the electron-deficient groups on the phenyl ring at the 4th position coupled smoothly with oxiranes to afford a moderate yield. The studies also showed that the larger substituents on the oxirane led to inhibition of the coordination of an oxygen atom to a palladium centre by steric hindrance, and no desired products were obtained (Scheme 24).^30^

**Scheme 25:**
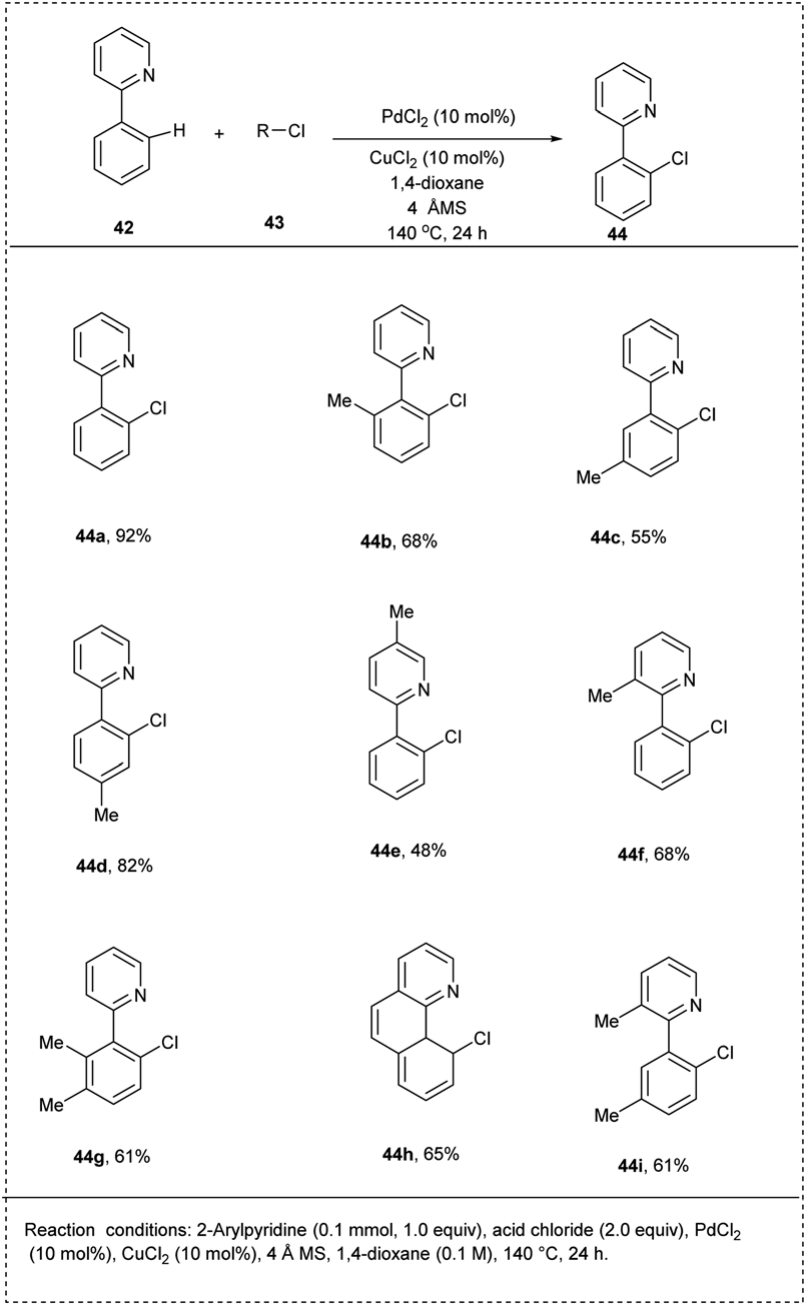
C–H chlorination of 2-phenyl pyridine with acid chlorides.

Jafarpour and coworkers 2014 disclosed a palladium-catalyzed protocol for the chelation-assisted C–H bond halogenation of arenes using acid chlorides as chlorinating agents. The protocol provided a greater scope as a mono-selective, straightforward, clean step for the synthesis of the aryl chlorides. The reaction was performed with 2-phenyl hydrazine and benzoyl chloride as substrates. This reaction utilised palladium chloride as a catalyst along with solvent copper(ii) chloride in 1,4-dioxane to afford an excellent yield of 92% of the expected product. The major advantage of this methodology was its excellent regioselectivity. This method was found to be the first palladium-catalyzed mono-selective *ortho* C–H chlorination of 2-phenyl pyridine. This method was found to be successful for the *ortho*-blocked 2-phenyl pyridine where the chlorination was carried out smoothly to afford a significant yield (Scheme 25).^31^

**Scheme 26:**
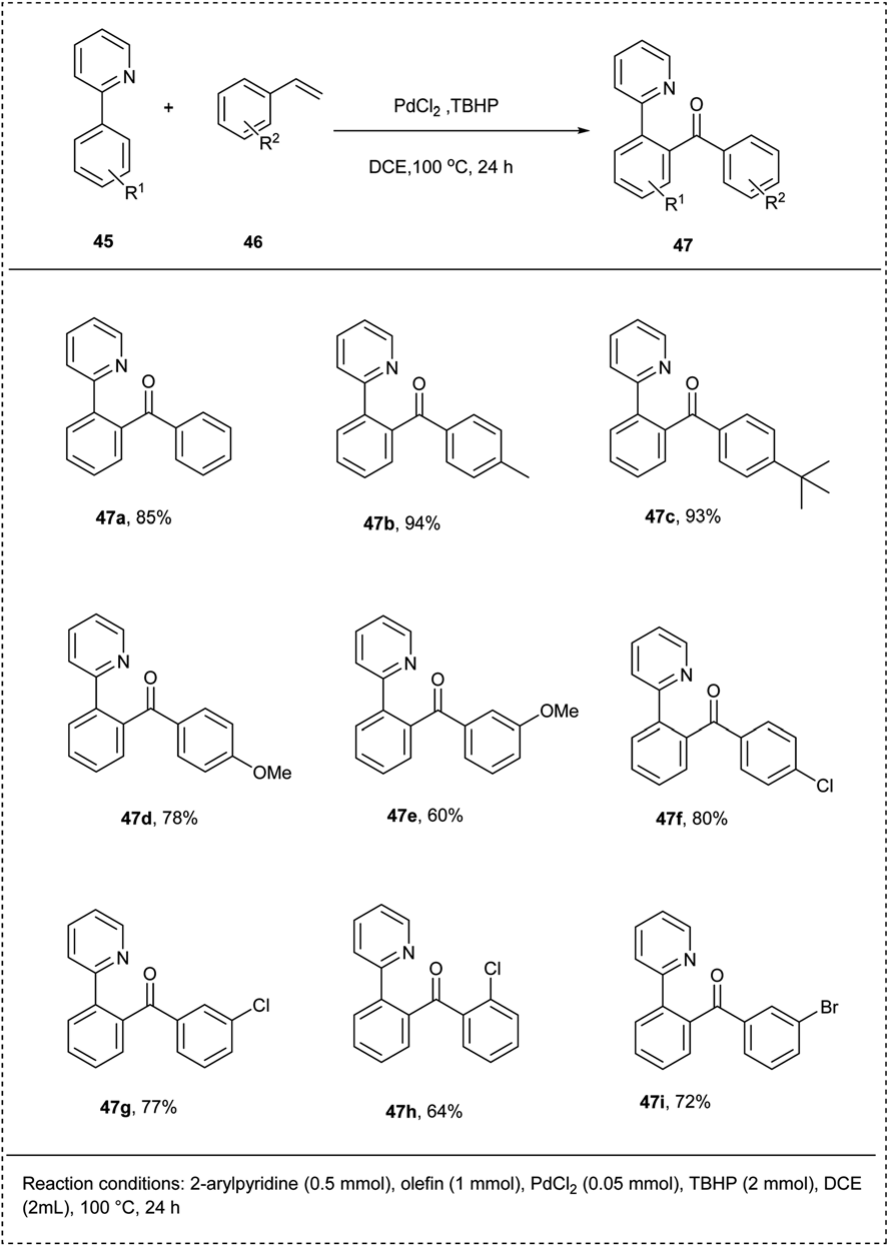
C–H activation by oxidative addition of arenes with olefins.

Bengara and coworkers in 2014 demonstrated an effective system for selective *ortho*-acylation of arenes by oxidative CH-bond activation using palladium as the catalyst. This protocol carried out the preliminary reaction using 2-phenyl pyridine and styrene as model substrates with PdCl_2_ as a catalyst. The important features of this reaction include high site selectivity and wide functional group tolerance. The reaction showed less efficiency under oxygen. This methodology utilized TBPB (*tert*-butyl per benzoate) as an oxidant and DCE as solvent. The reaction proceeded smoothly and gave an excellent yield of 90%. The studies showed no significant increase in the yield of the desired product when the temperature was raised. Better yields of the acylated products were found for the olefins with *para*-substituted electron-rich functional groups. The studies found that no reaction was carried out for the olefins with the electron-deficient functional groups (Scheme 26).^32^

**Scheme 27:**
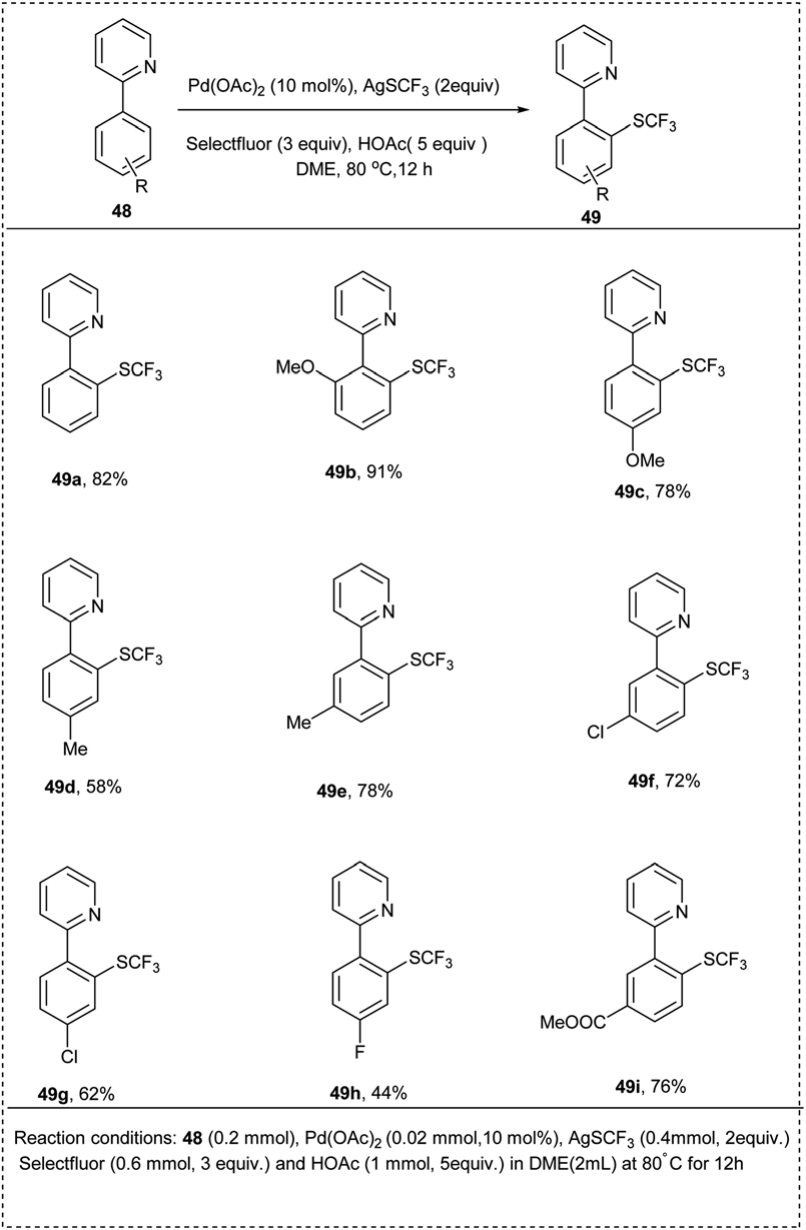
Direct C–H trifluoromethyl thiolation of 2-phenylpyridine.

In 2014, Huang put forward an efficient synthetic approach toward a palladium-catalyzed *ortho*-selective mono-trifluoromethyl thiolation reaction of arenes. 2-Phenyl pyridine along with AgSCF_3_ in the presence of Pd(OAc)_2_ was utilized for the reaction. The studies showed that the reaction proceeds through a ligand exchange pathway by using readily available silver trifluoromethyl thiolate (AgSCF_3_). The reaction utilized HOAc as an additive and solvent DME to afford the desired product with an excellent yield of 85%. The protocol was also found to be good toward substrates with an electron-donating group. The studies showed that acidic protons have no significant effect on the reaction. The studies disclosed that the electron-withdrawing group on the aromatic ring gave poor yield due to the strong coordinating effect of CN toward silver, which interfered with the ligand exchange. *Ortho*-methylpyridine afforded a low yield of the expected product due to the steric repulsion. Facile F to SCF_3_ ligand exchange ensured a smooth overall reaction. This method provides a greater strategy for the direct *ortho*-functionalization reactions using an oxidant and a nucleophilic reagent (Scheme 27).^33^

**Scheme 28:**
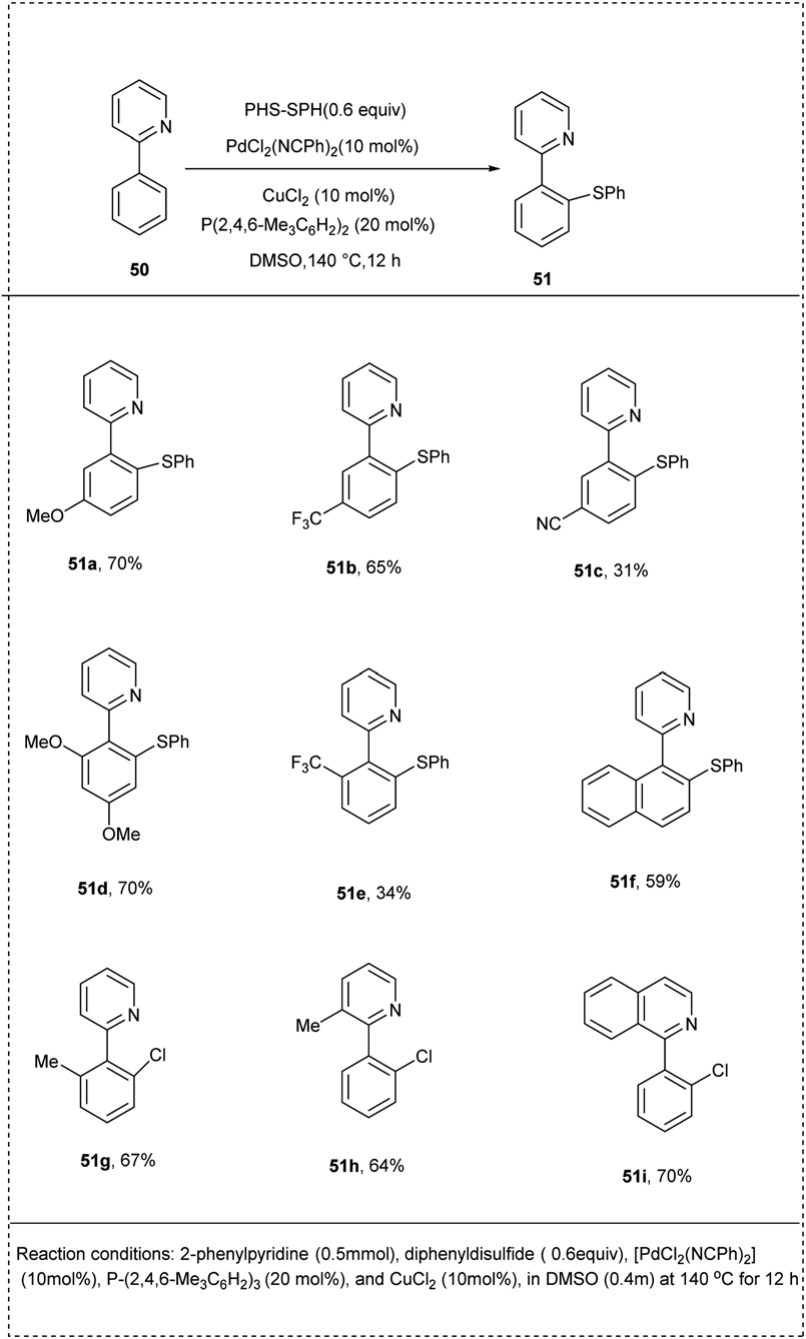
Direct C–H thiolation of 2-phenyl pyridine with diphenyl disulfide.

In 2014, Nishihara and coworkers introduced a simple and useful synthetic strategy for the direct thiolation of arenes having directing groups, with disulfides or thiols under a palladium catalyst. They carried out the preliminary reaction using 2-phenyl pyridine with diphenyl disulfide as a model substrate, and [PdCl_2_(NCPh)_2_] in DMSO was identified as the active catalyst of choice for this reaction. The addition of CuCl_2_ and phosphine ligands significantly enhanced the yield, achieving 87% of the desired product. The studies revealed that the use of phosphine ligands plays a crucial role in the dissociation of the palladium dimer to form the active monomeric species. The 2-phenyl pyridine possessed both electron-rich and electron-deficient groups at their *para* position afforded to desired products with good yield. The studies disclosed that the reaction proceeded with *ortho*-selectivity and induced a more significant yield of the desired product. This methodology provides a new pathway for preparing highly functionalized aryl sulfides in a more significant manner (Scheme 28).^34^

**Scheme 29:**
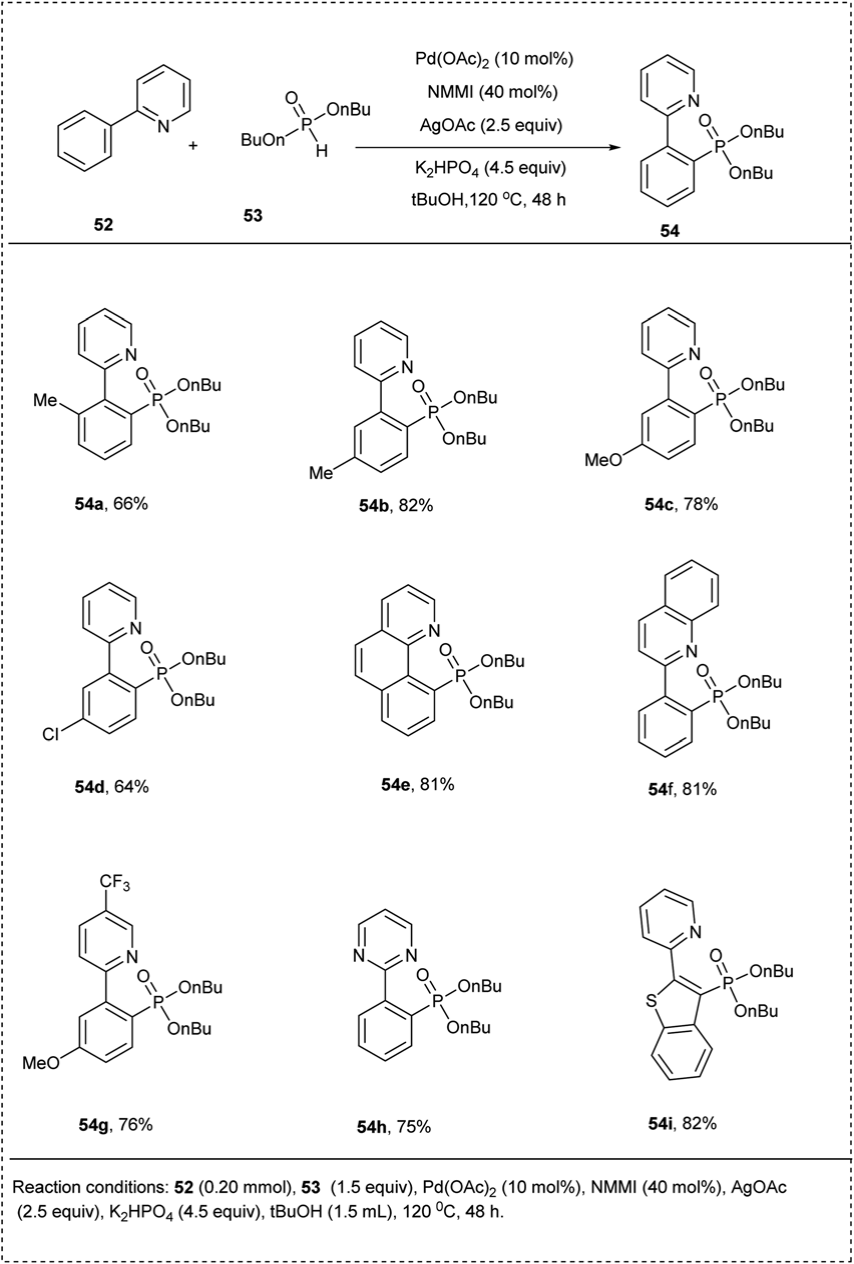
Direct phosphorylation of 2-phenyl pyridine.

In 2013, Murakami and co-workers elegantly developed a method for the phosphorylation of the C(sp^2^)–H bond for pyridine-directed groups using a palladium catalyst. The initial reaction was conducted by using 2-phenylpyridine and commercially available H-phosphonate in the presence of palladium(ii) acetate, *N*-methyl maleimide (NMMI), silver(i) acetate, and K_2_HPO_4_ to afford significant *ortho*-phosphonate product. They conducted extensive studies on the above-discussed strategy by using α-hydroxyalkyl phosphonate other than H-phosphate as the phosphorating reagent and obtained an excellent yield of 70%. The studies disclosed that *meta*-substituted functional groups on the substrate undergo phosphonate in the less steric hindered site to afford good yields. This methodology showed wide functional group tolerance and showed how α-hydroxyalkyl phosphonates can be used as covert phosphorating agents to prevent the catalyst from deactivating (Scheme 29).^35^

**Scheme 30:**
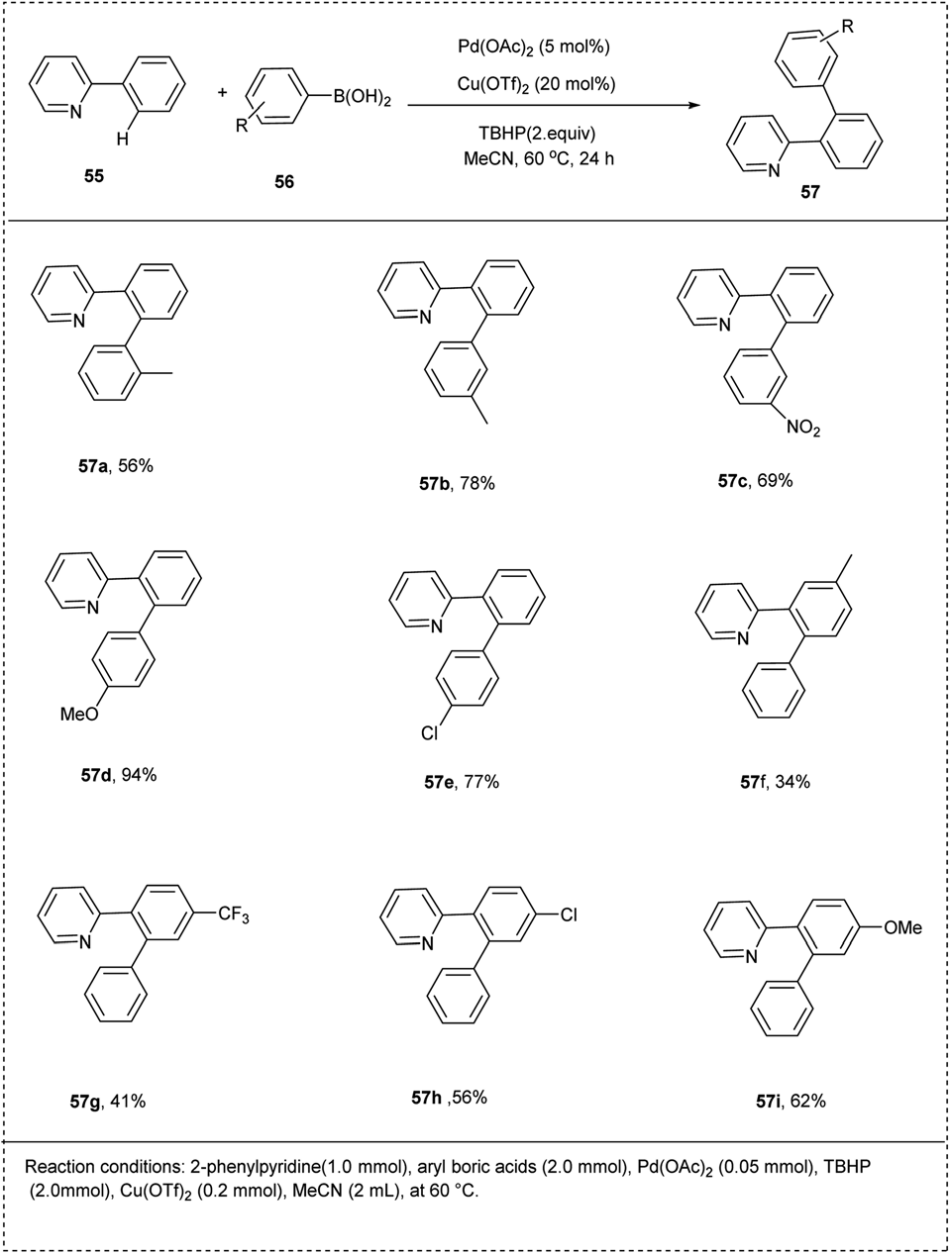
*Ortho* arylation of 2-phenylpyridine with aryl boric acid.

In 2013, Fu and co-workers designed a palladium-catalyzed protocol for the oxidative *ortho*-arylation of 2-phenyl pyridine. They carried out the preliminary reaction using 2-phenyl pyridine and aryl boric acid as a model substrate, and Pd(OAc)_2_ was identified as the active catalyst of choice for this reaction. They also utilized TBHP as an oxidant and Cu(OTf)_2_ as a co-oxidant for the couplings between 2-phenyl pyridines and aryl boric acids to afford an improved yield of about 94%. The studies disclosed that the temperature was found to be important for the selectivity of the reaction. However, for higher temperatures, the formation of disubstituted products and the undesired biphenyl products were found. A better yield of products was obtained for the electron-donating substrates compared to electron-withdrawing ones. The studies also showed that the substituents on the phenyl ring of the substrate, both the electron-rich and electron-deficient groups, lead to afford the desired product with poor yield because the substituent might interfere with the pyridine's ability to coordinate and the pyridine palladacycle's ability to form (Scheme 30).^36^

**Scheme 31:**
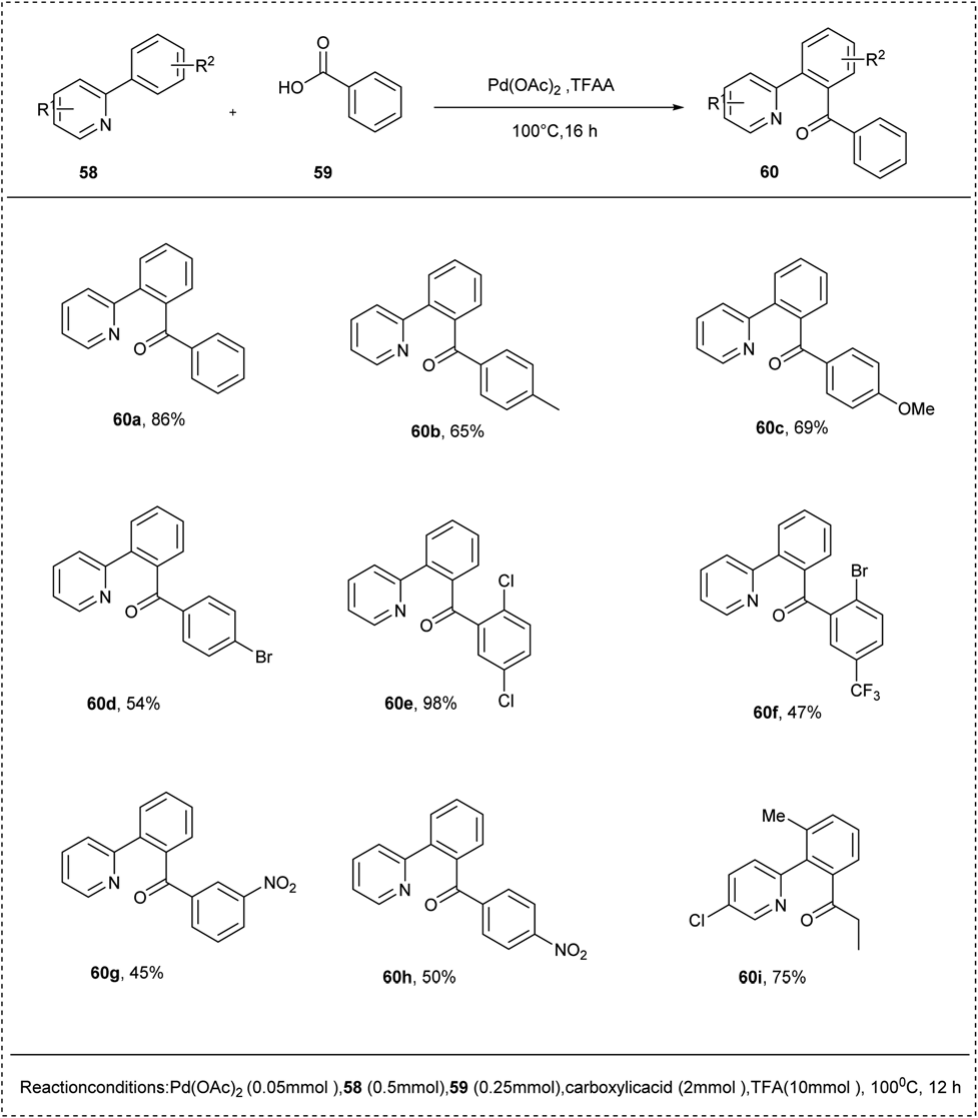
C–H acylation of 2-phenylpyridine with benzoic acid.

In 2013, Chai and his co-workers introduced the synthesis of aromatic ketones using an efficient palladium catalyst by selective aromatic CH bond acylation. The preliminary reaction was carried out using 2-phenyl pyridine and carboxylic acids as the acylating reagents. Palladium acetate showed high catalytic activity towards this reaction along with trifluoroacetic anhydride, which acts as an activated agent to afford an excellent yield of 82% of the desired product. The studies disclosed that electron-withdrawing groups show lower reactivity towards the reaction than the electron-rich functional groups. This reaction showed tolerance with various functional groups (Scheme 31).^37^

**Scheme 32:**
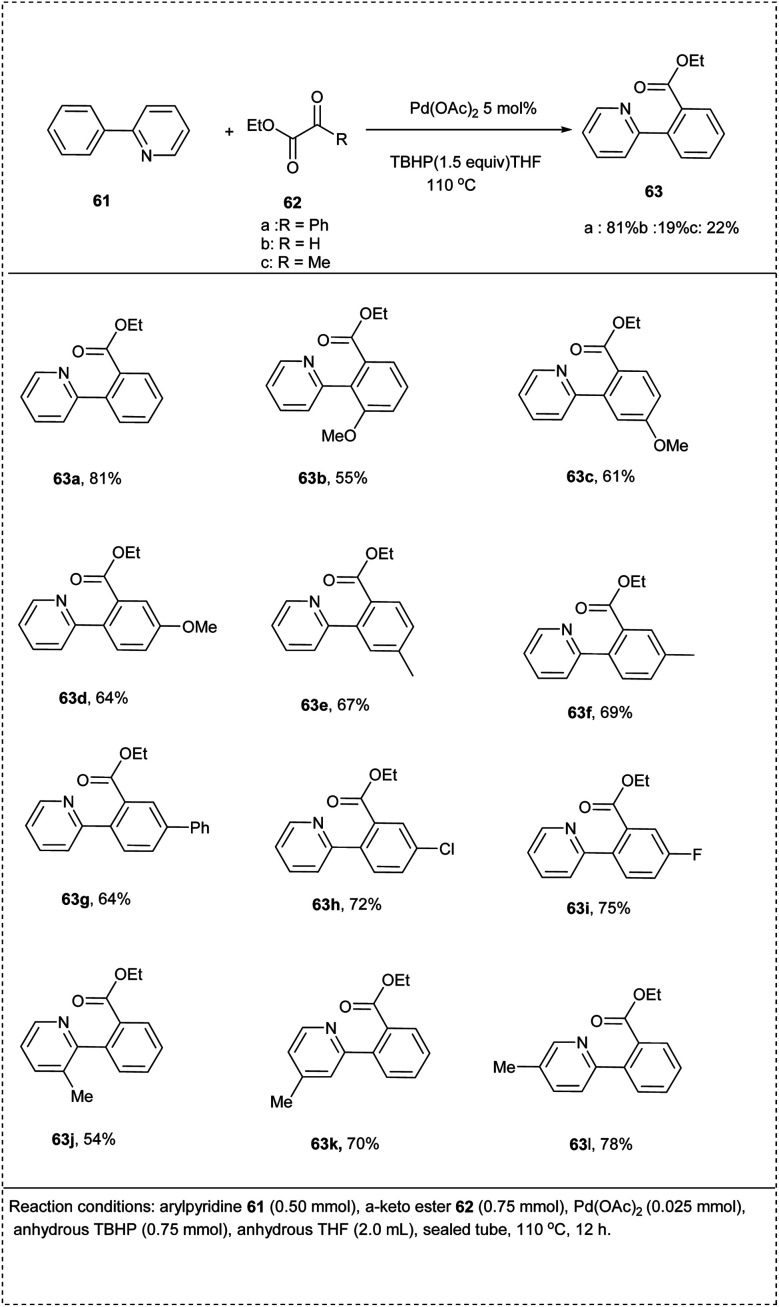
Direct alkoxy carbonylation of C–H bond of 2-phenylpyridine.

In 2013, Wang and his co-workers put forward a direct alkoxy carbonylation reaction using a palladium catalyst. This reaction includes 2-phenyl pyridine and ethyl benzoyl formate as model substrates. This reaction utilized Pd(OAc)_2_ as a catalyst under the condition of THF as solvent and *tert*-butyl hydroperoxide (TBHP) as an oxidant to afford a moderate yield of 81% of desired alkoxy carbonylation products. These reactions of aryl pyridines with a-keto esters indicated wide functional group tolerance and a broad scope of substrates with high selectivity. Electron-deficient groups on the substrate reacted smoothly to achieve the desired product in better yield than bearing electron-rich functional groups. The reaction also showed that substituents on the phenyl ring of 2-phenyl pyridine were well tolerated to undergo this reaction (Scheme 32).^38^

**Scheme 33:**
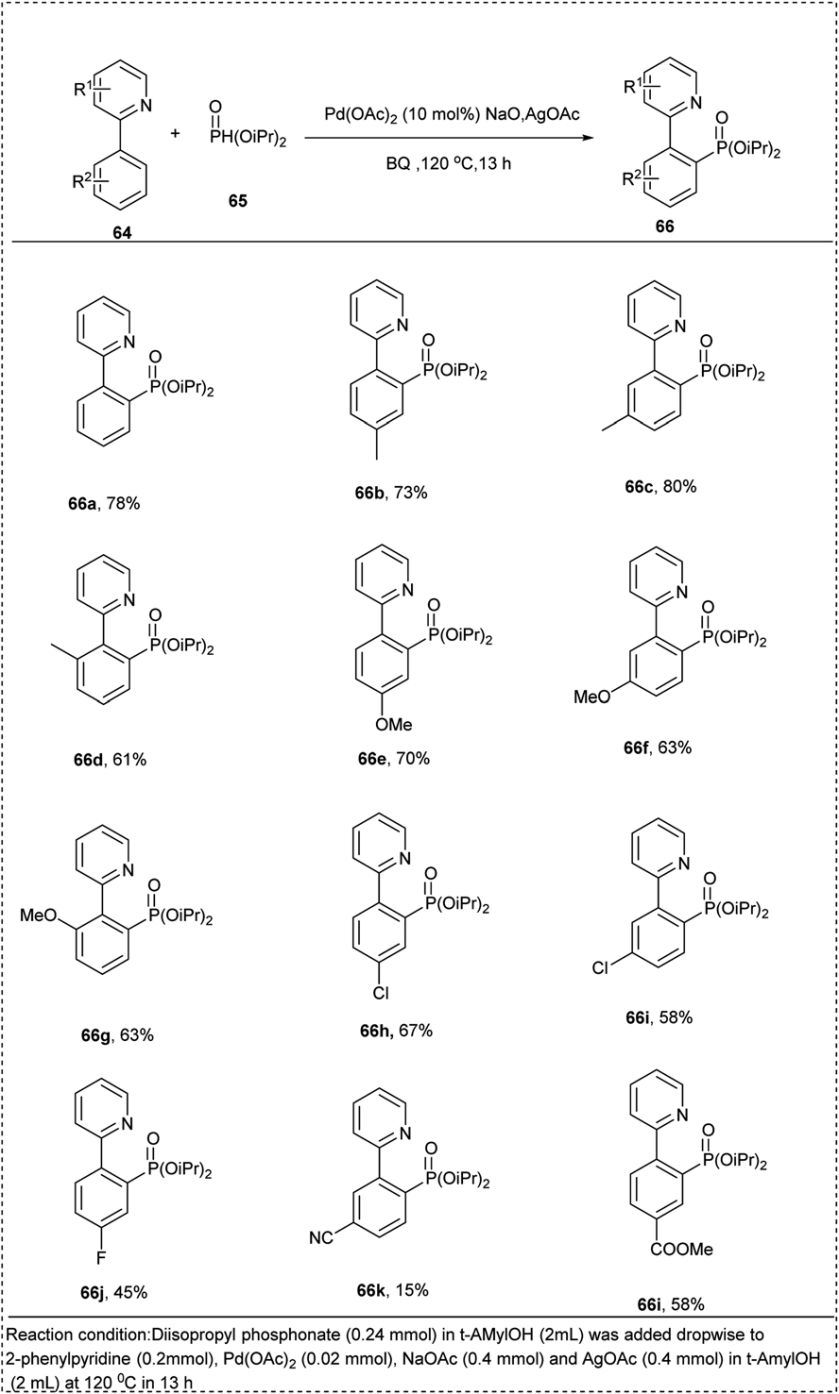
Palladium-catalyzed C–H phosphorylation of 2-phenylpyridine.

In 2013, Feng and his co-workers put forward a synthetic approach for the phosphorylation of aryl CH-bond using a palladium catalyst. This reaction proceeded using 2-phenyl pyridine with H-phosphonate added dropwise. This reaction utilized Pd(OAc)_2_ as a catalyst, AgOAc as an oxidant, NaOAc as a base, along with solvent *t*-AmylOH to afford the desired product of moderate yield with 84%. Further studies of reaction showed that the arenes with electron-donating groups in the *meta* and *para* positions tolerated well to the reaction better to achieve the desired product than the *ortho*-substituted arenes (Scheme 33).^39^

**Scheme 34:**
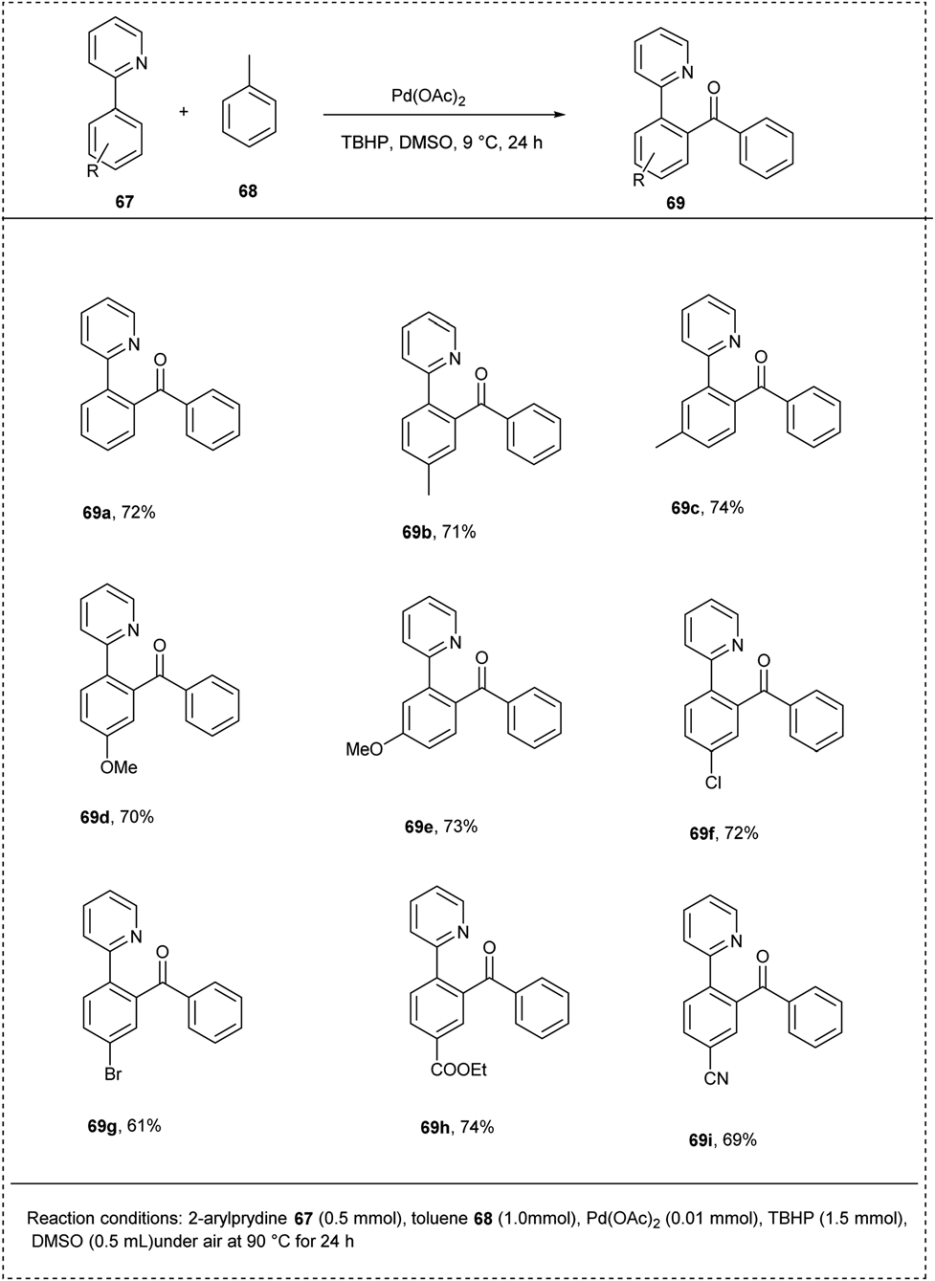
C–H acylation of 2-phenylpyridine with toluene derivatives.

In 2012, Sun achieved an attractive methodology for the direct *ortho*-acylation of 2-aryl pyridine under a palladium catalyst. This reaction utilized 2-phenyl pyridine, which undergoes oxidative coupling without pre-functionalized toluene derivatives to synthesize 2-pyridyldiaryl ketones. This method was found to be successful in developing a new synthetic approach by utilizing non-functionalized toluene as the acylation reagent. A better yield of about 74% was found to be obtained while utilizing Pd(OAc)_2_ as a catalyst along with TBHP as an oxidant. The studies disclosed that toluene can be compatible as an acylation reagent as well as a solvent for this benzoylation reaction. This method was found to be favourable for substrates with both electron-donating and electron-withdrawing substituents and led to a good yield of desired products. The major characteristics of this method include a wide substrate scope and high position selectivity (Scheme 34).^40^

**Scheme 35:**
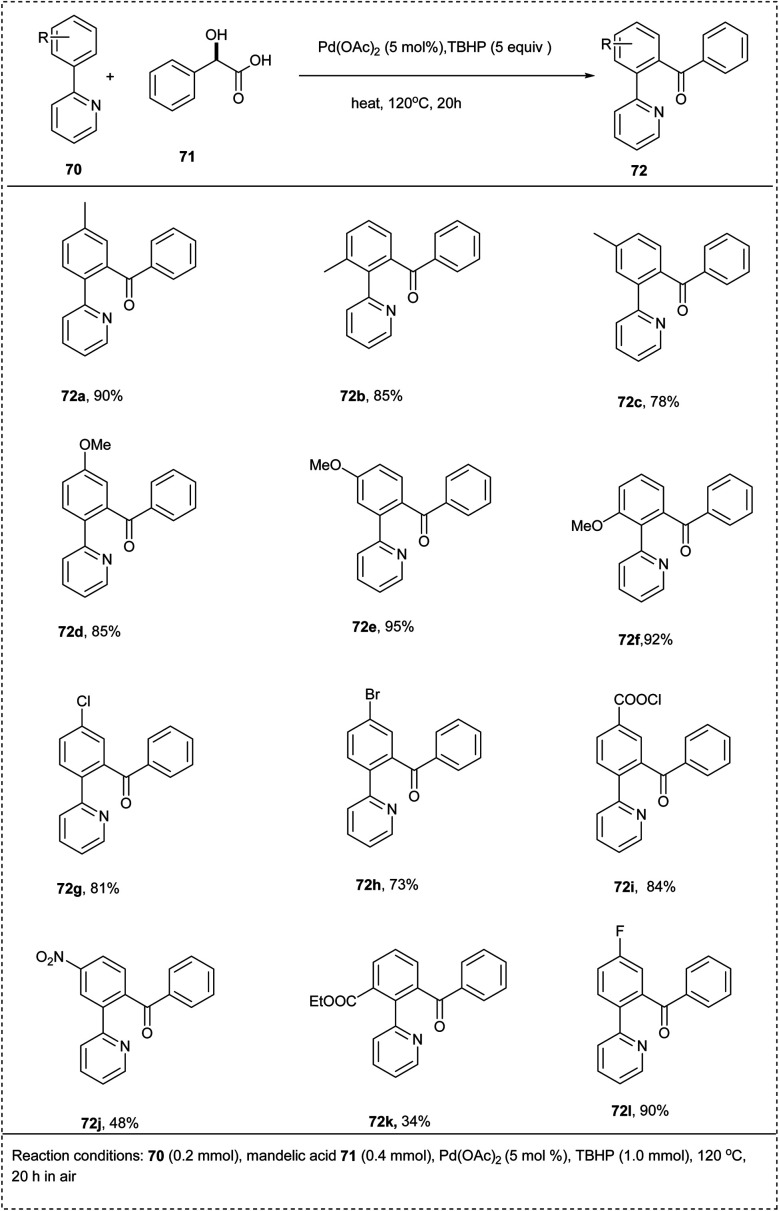
Decarboxylative acylation of arenes with mendalic acid derivatives.

In 2012, Liu and coworkers disclosed CH-activation with an efficient palladium-catalyzed decarboxylative acylation of arenes with mandelic acid derivatives. For this reaction, they found Pd(OAc)_2_ and *tert*-butyl hydroperoxide (TBHP) as the optimal catalyst and oxidant to afford a good yield of 82% of the desired carboacylation product. Both the electron-withdrawing and electron-donating of the phenyl group performed well in this protocol, affording the desired products in excellent yields. This reaction was found to be highly applicable, irrespective of the electronic nature of substrates. The major characteristic of this strategy was that it provides a convenient and economical way to synthesize aryl ketones (Scheme 35).^41^

**Scheme 36:**
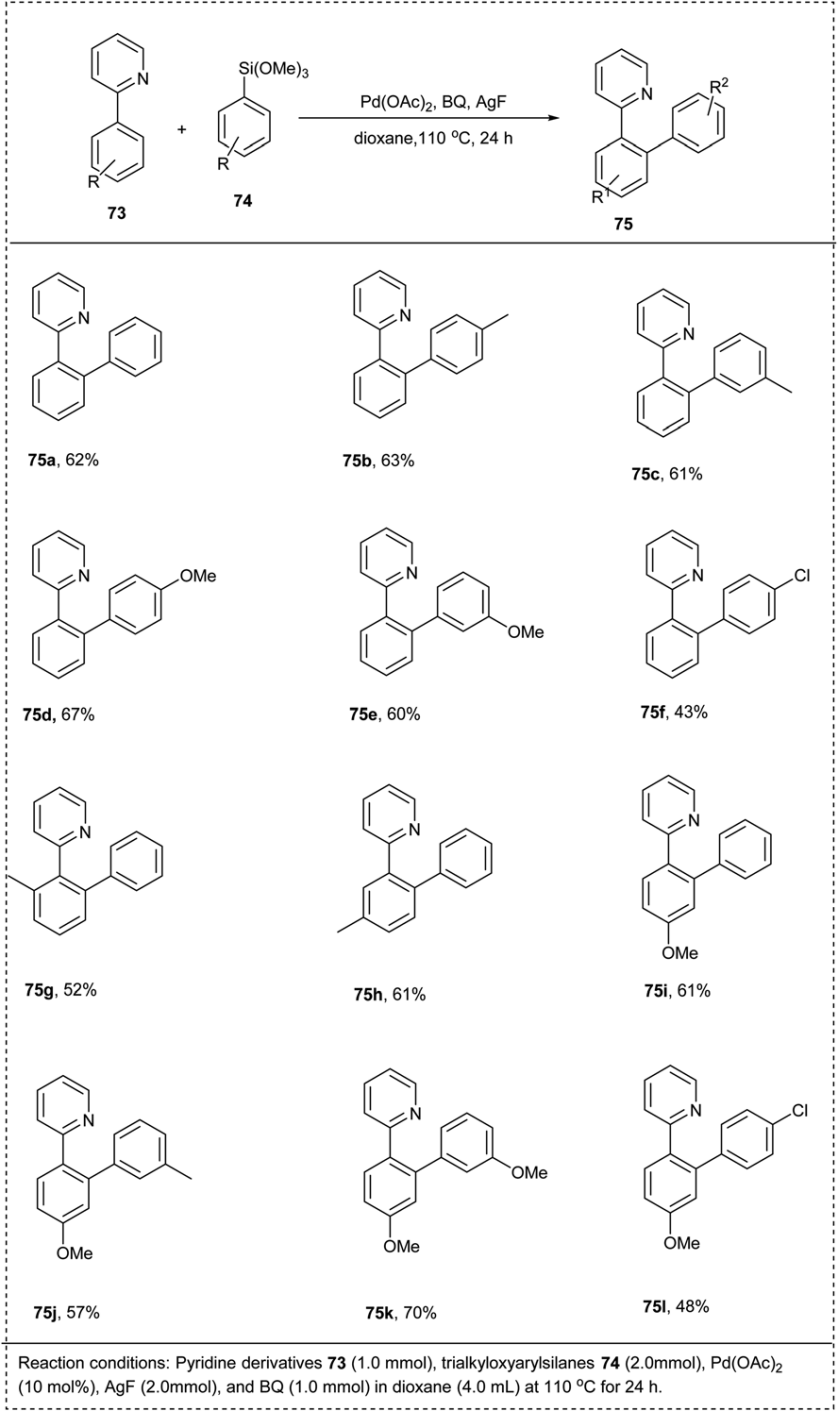
Direct *ortho*-C–H arylation of 2-phenylpyridine with aryltrimethoxysilane.

In 2011, Sun *et al.* introduced a simple and useful synthetic strategy for the cross-coupling reaction between 2-aryl pyridine and aryltrimethoxysilane using palladium catalyst for the direct *ortho*-CH-arylation. In this strategy, the nature of the oxidant, additive, and solvent, as well as the catalyst, played an essential role in the reaction efficiency. The studies disclosed Pd(OAc)_2_ showed excellent catalytic activity for this reaction along with AgF serves as both a fluoride source as well a co-oxidant with benzoquinone (BQ)to oxidize Pd(0)species back to Pd(ii), solvent dioxane and obtained a moderate yield of 62%. The electron-rich groups in *ortho*, *meta*, and *para* of the phenyl ring reacted smoothly to afford the desired product in a good yield, whereas the electron-deficient group on the phenyl ring resulted in no desired product. The major advantage of this methodology was its good regioselectivity, and no diarylated products or other byproducts were obtained (Scheme 36).^42^

**Scheme 37:**
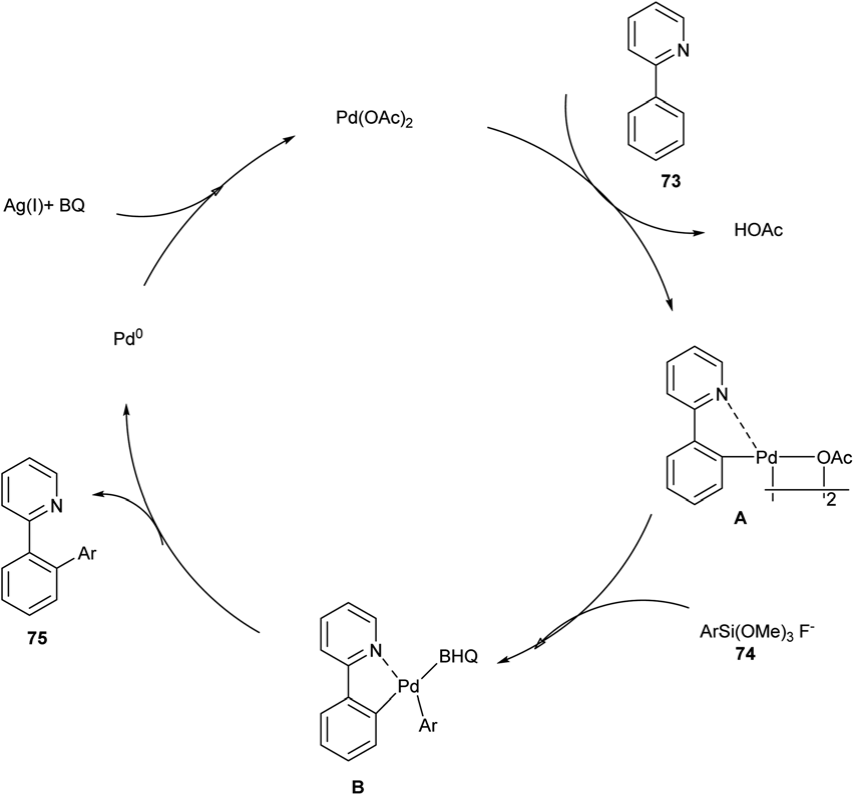
The plausible mechanism of the above reaction.

The mechanism involved in (Scheme 37) is initially 2-phenylpyridine reacted with Pd(OAc)_2_ and forms a cyclopalladated intermediate A which reacted with the *in situ* generated pentavalent silicate to form the (aryl)(2-phenylpyridine) palladium(ii) species B in which benzoquinone act as a ligand to coordinate with palladium. Further, through reductive elimination, liberates arylated 2-phenylpyridine and Pd(0) released was reoxidized by Ag(i) and *p*-benzoquinone to regenerate Pd(ii), which continued the catalytic cycle with high regioselectivity.^42^

**Scheme 38:**
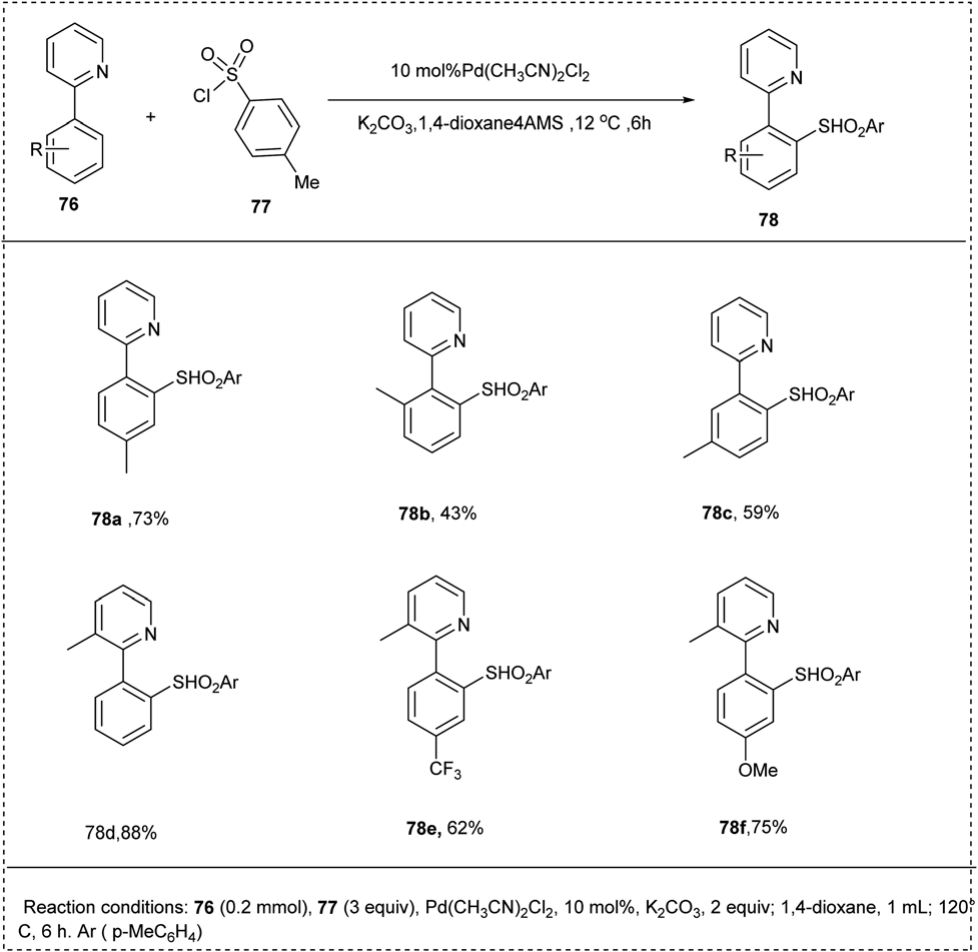
C–H sulfonation of 2-phenyl pyridine with sulfonyl chloride.

Dhong and coworkers demonstrated an efficient synthesis of sulfones from cross-coupling with aryl sulfonyl chlorides and aryl pyridine by employing a palladium-catalyzed CH-activation protocol in 2009. This reaction utilized 2-phenyl pyridine and *p*-tolyl sulfonyl chloride as model substrates and utilized Pd(CH_3_CN)_2_Cl_2_ as a catalyst along with K_2_CO_3_ in 1,4-dioxane, resulting in an excellent yield of 82%. This methodology developed a regioselective sulfone synthesis that did not require a pre-functionalized organometallic reagent. Substrate scope studies disclosed the efficient reaction of both *para*- and *meta*-substituted substrates to furnish the required product in good yields. The electron-donating groups on the substrate lead to significant yield, whereas electron-deficient groups diminished the yield of the desired product (Scheme 38).^43^

**Scheme 39:**
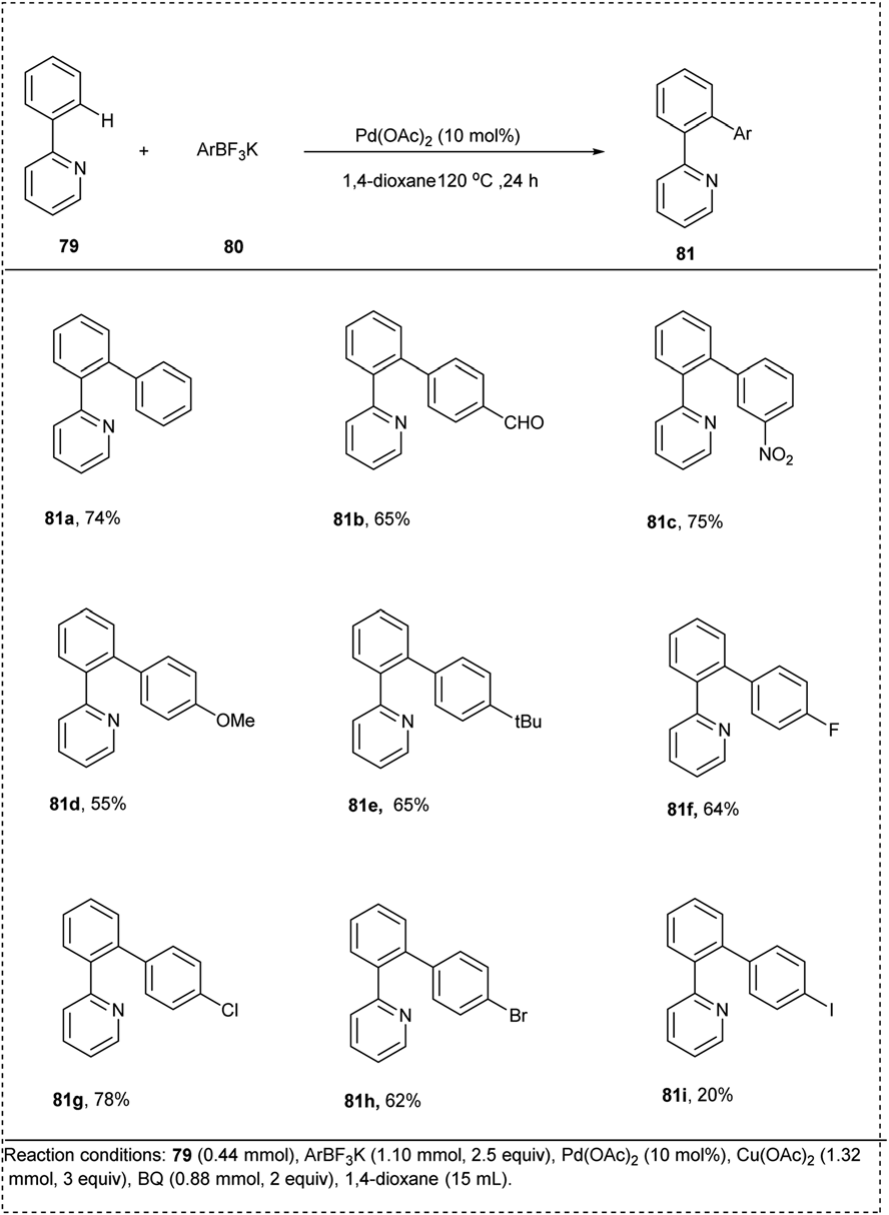
C–H functionalization by *ortho*-arylation of 2-phenyl pyridine with potassium aryl trifluoroborate.

In 2009, Wu and his coworkers put forward an efficient one-pot synthetic approach towards *ortho*-arylation through palladium-catalyzed CH-activation of 2-phenylpyridine and pyridine derivatives with potassium aryltrifluoroborates. They utilized palladium(ii) acetate as the coupling reagent and catalyst, and the studies disclosed that copper(ii) acetate and 1,4-dioxane were the optimal oxidant and solvent for this reaction. *p*-Benzoquinone was also utilized as a co-oxidant in the transmetalation–reductive elimination step. This reaction was found to be obtained in a good yield of 74%. The major drawback of this methodology was the use of excess amounts of oxidants [Cu(OAc)_2_ and BQ (Scheme 39)].^44^

**Scheme 40:**
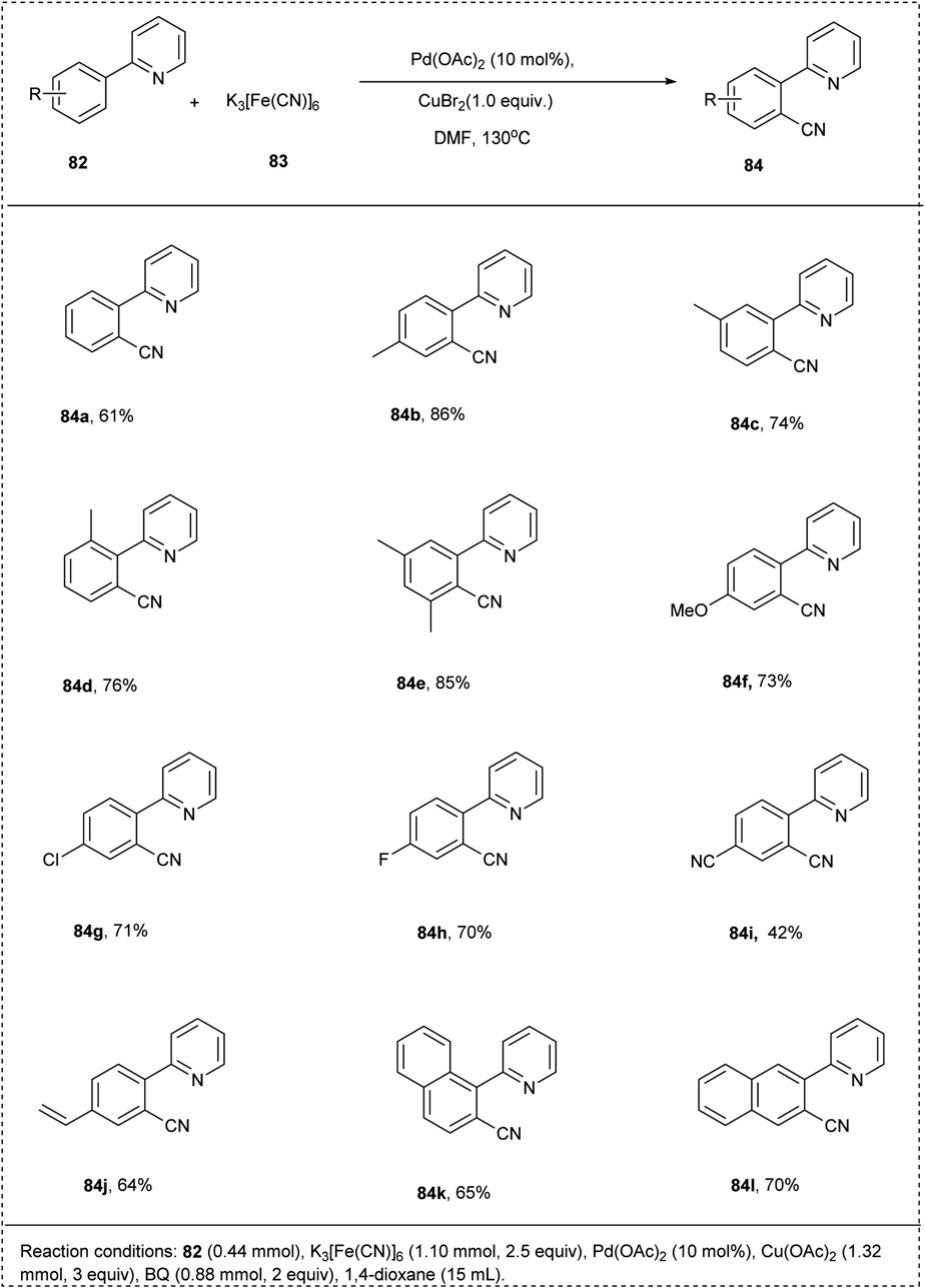
C–H cyanation of 2-phenyl pyridine using Pd catalyst.

In 2009, Cheng successfully achieved a chelation-assisted palladium-catalyzed methodology for the activation of the CH-bond by *ortho* cyanation, leading to the synthesis of aromatic nitrile. This reaction utilized 2-phenyl pyridine and K_3_[Fe(CN)]_6_ as a cyanating reagent. This cyanation reaction also utilized Pd(OAc)_2_ as a catalyst with Cu(OAc)_2_ and oxidant CuBr_2_ in DMF under air to afford an excellent yield of 81% of desired acylation products. The 2-arylpyridines, which possessed electron-rich arenes, reacted smoothly to achieve the required product in better yields than bearing electron-deficient functional groups. This methodology was found to be favourable for *meta*-substituted substrates proceeds *ortho*-cyanated products at the less steric hindered position with high regioselectivity and had significant yield. This strategy provides the synthesis of aromatic nitriles economically and conveniently (Scheme 40).^13^

**Scheme 41:**
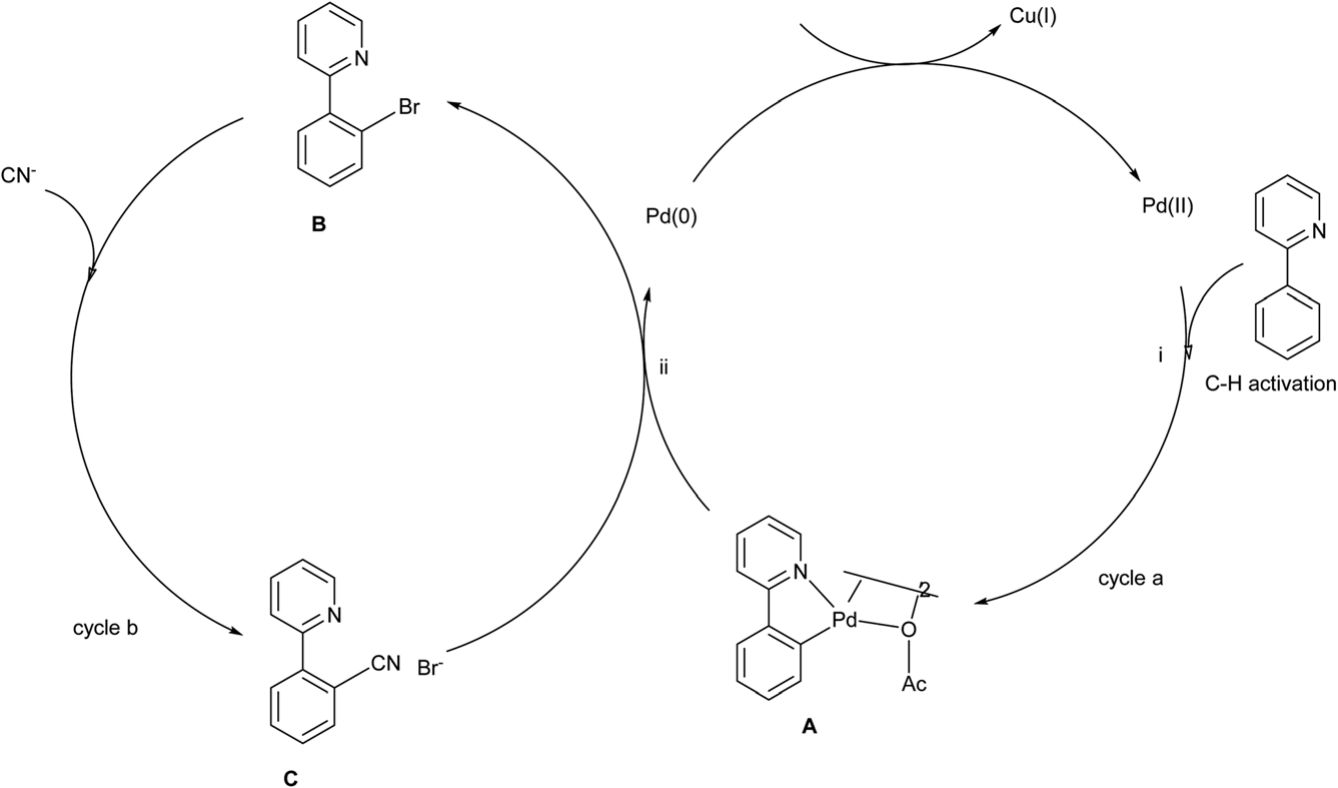
The plausible mechanism of the above reaction.

The proposed mechanism of (Scheme 41) involves the chelate-directed C–H activation of 2-phenyl pyridine and forms a cyclopalladium species A. Catalytic cycle of step ii involves the C–Br bond formation by reductive elimination and generates a bromination intermediate along with a Pd(0) species which undergoes oxidized to Pd(ii) by Cu(ii). In the cycle b cyanated product was generated by the catalysed process of palladium and copper.^13^

**Scheme 42:**
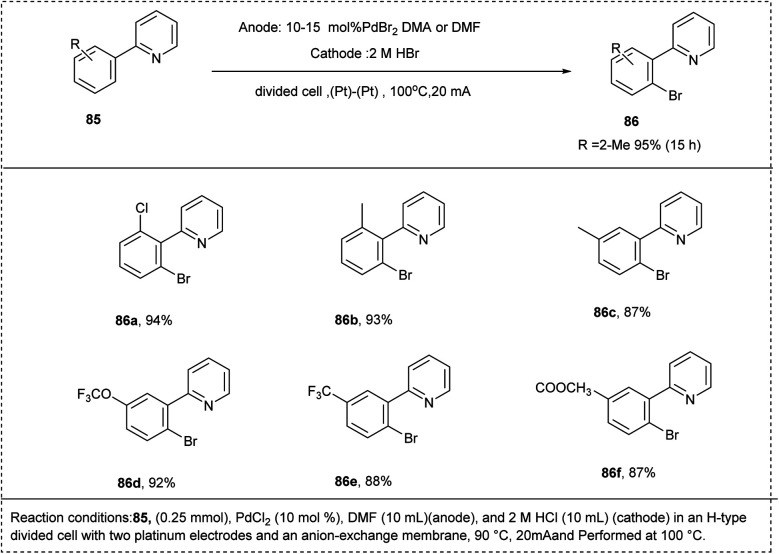
C–H halogenation of 2-phenyl pyridine with hydrogen halides by electrochemical oxidation.

In 2009, Kakiuchi and his co-workers developed an aromatic CH-halogenation under electrochemical oxidation conditions. The halogenation of the CH-bond was carried out using 2-phenyl pyridine along with hydrobromic acid using PdBr_2_ as a catalyst and solvent DMF to afford a good yield of 94%. The studies showed that both the electron-rich and electron-deficient functional groups were well tolerated in this reaction. The studies disclosed that the *meta*-substituted aryl pyridine undergoes selectively at the less hindered *ortho* position. The regioselectivity was controlled by tuning the electric current (Scheme 42).^45^

**Scheme 43:**
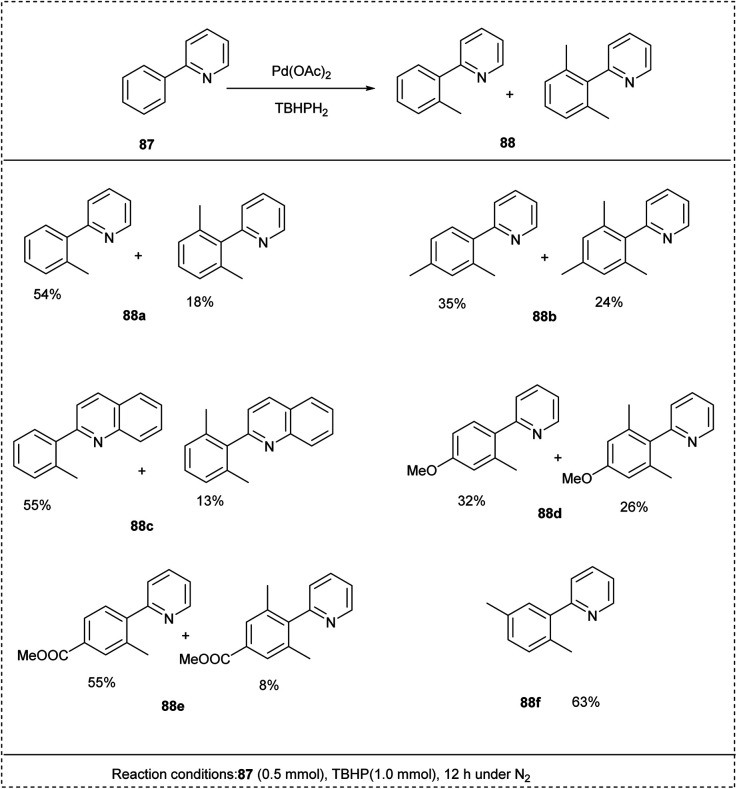
C–H methylation of 2-phenylpyridine using peroxides.

In 2007, Li introduced a novel method using peroxide compounds as methylating reagents and hydrogen acceptors for the direct methylation of aryl C–H bonds. The preliminary reaction was carried out by using 2-phenyl pyridine and *tert*-butyl peroxide, and Pd(OAc)_2_ as a catalyst. The reaction utilized *tert*-butylbenzene as an optimal solvent to afford an excellent yield of about 70%. The studies disclosed that the decrease in the temperature would affect the yield of the desired products. This methodology put forward a new synthetic approach for the direct alkylation of aryl C–H bonds by using alkyl radicals rather than organometallic reagents (Scheme 43).^46^

**Scheme 44:**
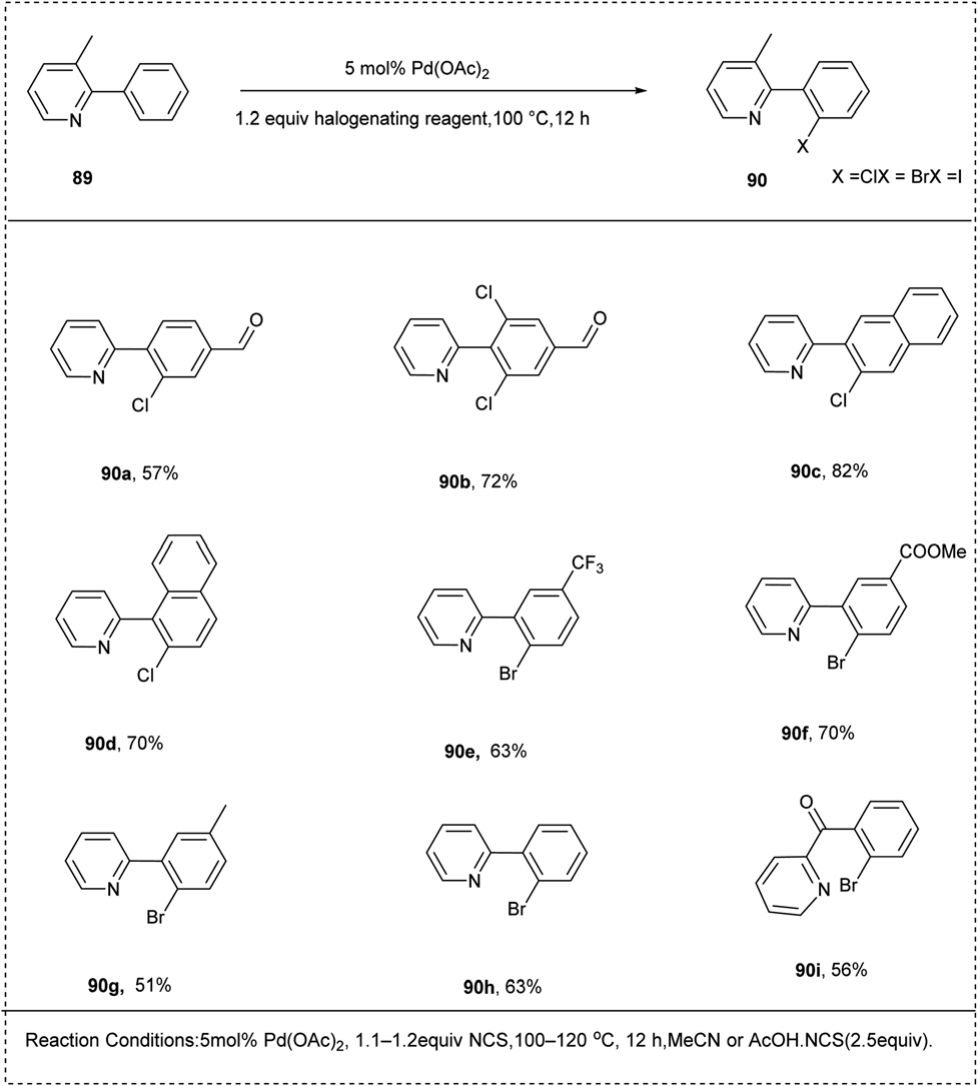
C–H halogenation of the arenes using electrophilic halogenating reagents.

In 2006, Sanford *et al.* explored the palladium-catalysed halogenation of the arenes using electrophilic halogenating reagents in AcOH and MeCN. They utilized 2-phenyl pyridine along with *N*-halo succinimides as a superior oxidant and obtained a yield of 79%. The studies showed that the arenes-bearing electron-deficient groups undergo halogenation to afford the desired product in a good yield. The studies also disclosed that the *meta*-substituted arenes undergo halogenation with high selectivity. The major characteristics of this methodology were its wide substrate scope and high functional group tolerance (Scheme 44).^47^

## 3. Conclusion and outlook

Over the past decade, C–H activation has emerged as an increasingly efficient tool in molecular science. This review encapsulates the advancements in palladium-catalyzed C–H activation. Transition metal-catalyzed C–H bond activation is an alternative to traditional cross-coupling reactions, minimising the lengthy synthetic routes to activate the coupling partners. The studies disclosed that Pd(OAc)_2_ was the most promising catalytic precursor to establish Pd-catalytic complexes and led to a significant product yield. Silver salts are usually used as an optimal oxidant for reacting. Some of the reported methods are highly promising as they exhibit excellent regioselectivity and stereoselectivity. Both electron-donating and electron-withdrawing groups showed exceptional functional group tolerance with a significant yield percentage. The usage of a Pd-catalysed reaction enabled to overcome the classical C–H bond activation challenges like high bond dissociation energy (BDE), lack of the active HOMO or LUMO to interact with transition metal catalytic centres, and difficulty to control the selectivity of C–H transformations. Direct carboxylation, amination, C–C bond formation, alkoxylation, C–X bond formation and fluorination of C–H bonds were easily achievable with transition metal catalyses. Recent reports suggested the utilisation of Pd-based catalysts in various polycyclic aromatic hydrocarbons (PAHs) and polycyclic heteroaromatics (PCHs) with different conformational differences. Exploring these avenues could lead to innovative methodologies that capitalize on the unique features of Pd(iv) in C–H activation. While the development of synthetically valuable catalytic cycles that employ C–H activation at Pd(iv) is still in its early stages, there is significant potential for unique selectivity in various molecular transformations.

## Data availability

No primary research results, software or code have been included and no new data were generated or analysed as part of this review.

## Conflicts of interest

There are no conflicts to declare.
